# HIV Drugs Inhibit Transfer of Plasmids Carrying Extended-Spectrum β-Lactamase and Carbapenemase Genes

**DOI:** 10.1128/mBio.03355-19

**Published:** 2020-02-25

**Authors:** Michelle M. C. Buckner, M. Laura Ciusa, Richard W. Meek, Alice R. Moorey, Gregory E. McCallum, Emma L. Prentice, Jeremy P. Reid, Luke J. Alderwick, Alessandro Di Maio, Laura J. V. Piddock

**Affiliations:** a Institute of Microbiology & Infection, College of Medical & Dental Sciences, University of Birmingham, Edgbaston, United Kingdom; b Institute of Microbiology & Infection, School of Biosciences, University of Birmingham, Edgbaston, United Kingdom; c Birmingham Advanced Light Microscopy, School of Biosciences, University of Birmingham, Edgbaston, United Kingdom; University of British Columbia

**Keywords:** antimicrobial resistance, *E. coli*, *K. pneumoniae*, ESBL, carbapenemases, KPC, CTX-M, carbapenemase

## Abstract

Antimicrobial-resistant (AMR) infections pose a serious risk to human and animal health. A major factor contributing to this global crisis is the sharing of resistance genes between different bacteria via plasmids. The WHO lists *Enterobacteriaceae*, such as Escherichia coli and Klebsiella pneumoniae, producing extended-spectrum β-lactamases (ESBL) and carbapenemases as “critical” priorities for new drug development. These resistance genes are most often shared via plasmid transfer. However, finding methods to prevent resistance gene sharing has been hampered by the lack of screening systems for medium-/high-throughput approaches. Here, we have used an ESBL-producing plasmid, pCT, and a carbapenemase-producing plasmid, pKpQIL, in two different Gram-negative bacteria, E. coli and K. pneumoniae. Using these critical resistance-pathogen combinations, we developed an assay using fluorescent proteins, flow cytometry, and confocal microscopy to assess plasmid transmission inhibition within bacterial populations in a medium-throughput manner. Three compounds with some reports of antiplasmid properties were tested; chlorpromazine reduced transmission of both plasmids and linoleic acid reduced transmission of pCT. We screened the Prestwick library of over 1,200 FDA-approved drugs/compounds. From this, we found two nucleoside analogue drugs used to treat HIV, abacavir and azidothymidine (AZT), which reduced plasmid transmission (AZT, e.g., at 0.25 μg/ml reduced pCT transmission in E. coli by 83.3% and pKpQIL transmission in K. pneumoniae by 80.8% compared to untreated controls). Plasmid transmission was reduced by concentrations of the drugs which are below peak serum concentrations and are achievable in the gastrointestinal tract. These drugs could be used to decolonize humans, animals, or the environment from AMR plasmids.

## Abstract (precis)

More and more bacterial infections are becoming resistant to antibiotics. This has made treatment of many infections very difficult. One of the reasons this is such a large problem is that bacteria are able to share their genetic material with other bacteria, and these shared genes often include resistance to a variety of antibiotics, including some of our drugs of last resort. We are addressing this problem by using a fluorescence-based system to search for drugs that will stop bacteria from sharing resistance genes. We uncovered a new role for two drugs used to treat HIV and show that they are able to prevent the sharing of two different types of resistance genes in two unique bacterial strains. This work lays the foundation for future work to reduce the prevalence of resistant infections.

## INTRODUCTION

The threat of untreatable infections due to antimicrobial resistance (AMR) has been recognized by global agencies, including the World Health Organization (WHO), and national governments (1–3). A key factor contributing to AMR is mobile genetic elements such as plasmids, which can carry multiple antimicrobial resistance genes (ARGs) and are commonly found within the order *Enterobacteriales* (which consists of one family, *Enterobacteriaceae*), including many difficult-to-treat species such as multidrug-resistant (MDR) Escherichia coli and Klebsiella pneumoniae. In particular, plasmids increasingly carry ARGs coding for proteins such as extended-spectrum β-lactamases (ESBLs; e.g., CTX-M), carbapenemases (e.g., KPC), and colistin resistance proteins (e.g., MCR-1) (4–6). The WHO lists carbapenem-resistant and ESBL-producing *Enterobacteriaceae* as a critical priority for which new drugs are needed (7), and the CDC lists them as an urgent and serious threat, respectively (8). Therefore, finding new strategies to combat ESBL- and carbapenemase-producing *Enterobacteriaceae* is of the utmost importance.

These bacteria have proven particularly difficult to treat for a few key reasons; one is that the genes coding for ESBLs and carbapenemases are most often carried on plasmids, most of which are conjugative. These AMR plasmids and ARGs are found within, and shared between, bacteria colocalizing the same niche, such as intestinal tracts (9–11); environments, including wastewater and river sediments (12); agricultural soil (13–15); and hospital surfaces (16, 17). Furthermore, ARGs frequently located on plasmids have been acquired by people traveling to areas of the world with high levels of AMR (18–20). With the single acquisition of an MDR, ESBL- or carbapenemase-producing plasmid, *Enterobacteriales* can go from being easily treatable to extremely challenging. Therefore, AMR plasmids pose a serious risk to human and animal health.

One example of a highly successful AMR plasmid is the IncK plasmid pCT, which carries a group 9 *bla*_CTX-M-14_ ESBL gene (5). pCT-like elements have been found in commensal and extraintestinal pathogenic E. coli isolates from humans and animals (21, 22). The CTX-M family is the largest group of ESBLs (23). *bla*_CTX-M-14_ is the most commonly found ESBL gene in parts of Asia and Spain (23). IncK plasmids in particular have contributed to the spread of *bla*_CTX-M-14_ in Spain (24) and the United Kingdom (25). The high rate of pCT conjugation and high degree of stability have contributed to the spread of pCT-like plasmids among diverse E. coli strains (21, 26, 27). E. coli from the ST131 clonal group is globally the most predominant cause of extraintestinal E. coli infections, such as urinary tract infections (UTIs), and has been associated with the success of some *bla*_CTX-M_ genes such as *bla*_CTX-M-15_ (23, 28, 29).

Likewise, the carbapenemase-producing plasmid pKpQIL and its variants have been isolated around the world in a variety of species but are predominantly found in K. pneumoniae (30–33). The prevalence and spread of pKpQIL in the United Kingdom have been well characterized (6, 34–38). pKpQIL is a 114-kb, IncFIIK2 plasmid carrying the *bla*_KPC_ carbapenemase gene, the *bla*_TEM_ β-lactamase gene, and heavy metal resistance and is self-transmissible (30, 39, 40). pKpQIL is well adapted to the K. pneumoniae host and is stably maintained in K. pneumoniae populations (31, 38).

One approach to tackling ESBL- and carbapenemase-producing *Enterobacteriales* is to reduce the prevalence of AMR plasmids, by decolonization of people, animals, and/or the environment. Antiplasmid compounds can act by reducing plasmid stability, resulting in eradication of plasmids from a population (termed plasmid curing), and/or preventing transmission of a plasmid to a new host (41, 42). Except for a few such as chlorpromazine, ascorbic acid, and linoleic acid, most compounds identified since the 1960s with reported antiplasmid activity are toxic to humans (42). Chlorpromazine belongs to the phenothiazine class of antipsychotic drugs (43, 44). However, the literature regarding the plasmid curing ability of chlorpromazine is controversial, and there are inconsistent reports of activity. Ascorbic acid (vitamin C) cured Gram-positive bacteria (Staphylococcus aureus and Pediococcus acidilactici) of some drug resistance plasmids (45–47). However, the impact upon clinically relevant Gram-negative bacterium-plasmid host combinations is unclear. The unsaturated fatty acid linoleic acid inhibited conjugation of some plasmids in E. coli (48) by inhibiting the activity of the TwrD ATPase (VirB11 homologue), which is involved in the conjugative machinery required for transmission (49). However, none of these compounds have been tested on pCT or pKpQIL.

In order to discover safe and efficacious ways to remove and/or prevent the spread of ESBL- and carbapenemase-producing plasmids from *Enterobacteriales*, we have developed a method to monitor transmission of pCT and pKpQIL in real time using fluorescent protein genes on both the plasmids and host strains E. coli ST131 (50–52) and K. pneumoniae Ecl8 (38, 53, 54). Plasmid dynamics within the bacterial population were monitored using flow cytometry and microscopy. We demonstrate that chlorpromazine reduced pCT and pKpQIL transmission, while linoleic acid inhibited only pCT, and ascorbic acid had little impact. This assay was then used in a medium-throughput screen (MTS) of the Prestwick FDA-approved library. We identified a novel role as antiplasmid compounds for two FDA-approved anti-HIV drugs, abacavir and azidothymidine (AZT; also called zidovudine), which reduced transmission of both the ESBL- and carbapenemase-producing plasmids in E. coli and K. pneumoniae. We also demonstrated the activity of these novel antiplasmid compounds at clinically achievable concentrations and at which they do not impact bacterial growth, and so the likelihood of selective pressure for the emergence of transmission-inhibitor resistance is minimal.

## RESULTS

### Development of assay to measure plasmid transmission in a bacterial population.

In order to rapidly measure the dynamics of pCT and pKpQIL transfer to host strains, fluorescence-based reporters were constructed in E. coli ST131 EC958 (clade C) and K. pneumoniae Ecl8, respectively. Prior to insertion of pCT into ST131 EC958, the resident plasmid (pEC958) (55) was removed using an incompatibility-based system (for additional detail, see Text S1 and Fig. S1a in the supplemental material).

10.1128/mBio.03355-19.1 TEXT S1 Supplemental results and methods. Download Text S1, DOCX file, 0.04 MB. Copyright © 2020 Buckner et al. 2020 Buckner et al. This content is distributed under the terms of the Creative Commons Attribution 4.0 International license.

10.1128/mBio.03355-19.2 FIG S1 (a) Curing E. coli ST131 EC958 of plasmid pEC958. Lane 1, Hyperladder VI Bioline (band size from the top: 48.5, 38.42, 33.5, 29.95, 24.51, 23.99, 17.05, 15, 12.14, and 10.09 kb). Lane 2, E. coli reference strain NCTC 50192 containing 147-, 63-, 36-, and 7-kb plasmids. Lane 3, E. coli ST131 EC958. Lane 4, E. coli ST131 EC958 cured of plasmid pEC958. Lane 5, Hyperladder 1-kb Bioline (band size from the top: 10, 8, 6, 5, 4, 3, 2.5, 2, and 1.5 kb). (b) Quantification of cells colocalized, as percentage of total cells in field of view, where colocalization is defined as pixels with both red and green signal, representing cells in very close contact, or where an individual cell expresses both fluorescent proteins. Data from 3 independent experiments. Error bars represent standard deviation from the mean. Download FIG S1, DOCX file, 2.0 MB. Copyright © 2020 Buckner et al. 2020 Buckner et al. This content is distributed under the terms of the Creative Commons Attribution 4.0 International license.

**FIG 1:**
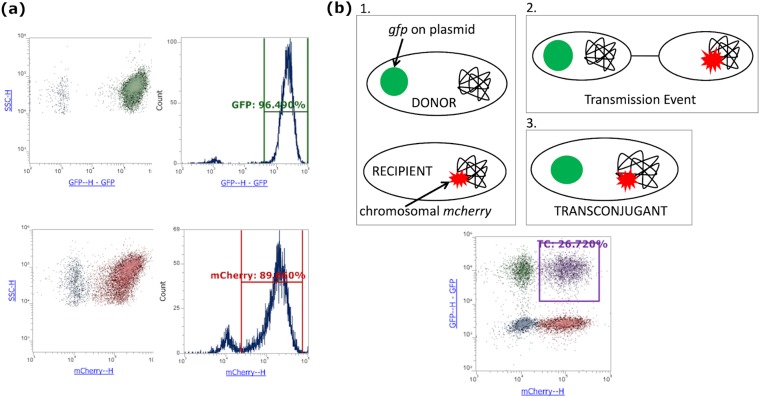
(a) Representative flow cytometry plots of ST131c EC958 pCT*gfp* (top) and ST131c EC958 *mcherry* (bottom). For each, 10,000 bacterial events were collected and are displayed in each plot. (b) Diagrammatic representation of transmission detection system using fluorescent markers. (Panel 1) Donor strains express *gfp* constitutively from the plasmid, while recipient strains express *mcherry* constitutively from the chromosome. (Panel 2) Conjugative transmission event occurs between donor and recipient strains. (Panel 3) Newly formed transconjugant expresses both *gfp* (plasmid) and *mcherry* (chromosome). (Bottom) Transmission experiment 24 h after combination of donor and recipient bacteria, with transconjugant bacteria (TC) indicated in purple square gate.

The *gfp* gene was inserted into pCT_CTX-M-14_, giving pCT*gfp*, which was transferred by conjugation into ST131c, forming ST131c pCT*gfp*. In order to monitor pKpQIL in K. pneumoniae, the *gfp* gene was inserted into the *bla*_KPC_ locus in pKpQIL, thus disrupting the *bla*_KPC_ gene and resulting in pKpQIL*gfp*. Conjugation was used to insert pKpQIL*gfp* into K. pneumoniae strain Ecl8, forming Ecl8 pKpQIL*gfp*. These strains formed the plasmid donors. The recipient strains were constructed by inserting *mcherry* into the *putPA* intergenic region, as per reference 56, in the chromosome of ST131c, thus forming ST131c *mcherry*, and of Ecl8, forming Ecl8 *mcherry*. Strains were confirmed by PCR and DNA sequencing (see Text S1 and Tables S4 to S6), and flow cytometry was used to confirm expression of fluorescent proteins (Fig. 1a). Plasmid transmission was measured by flow cytometry, which quantified the number of green fluorescent protein (GFP)-positive bacteria (donors), mCherry-positive bacteria (recipients), and GFP-positive/mCherry-positive bacteria (transconjugants) (Fig. 1b).

**FIG 2:**
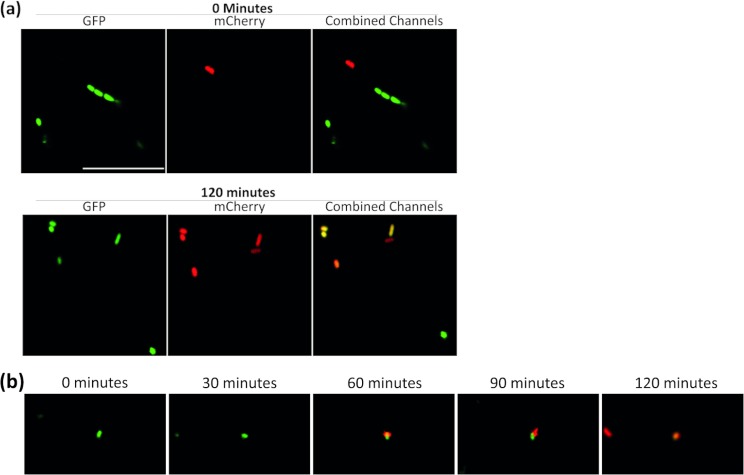
Confocal microscopy images of pCT transmission in MOPS minimal medium. (a) pCT transmission at time zero and 120 min. (b) Microscopy time course, showing a GFP^+^ bacterium interacting with an mCherry^+^ bacterium 60 and 90 min after mixing; then, at 120 min the GFP^+^/mCherry^+^ bacterium is present.

Since fluorescent proteins were used as an indicator of plasmid transmission, confocal microscopy was used to monitor plasmid transmission. Images of E. coli/pCT*gfp* donor and E. coli *mcherry* recipient strains were collected at time zero, prior to transmission (Fig. 2a, top panel), and at 120 min, which was sufficient time for transmission events to occur and with transconjugant bacteria clearly visible (Fig. 2a, bottom panel). A time course was performed with the same strains by taking images every 10 min up to 120 min. During this time lapse, donor and recipient bacteria coming into close contact were captured at 60 and 90 min, as well as a transconjugant bacterium expressing both green and red fluorescent proteins at 120 min (Fig. 2b). Over 120 min, the number of colocalization events (defined as GFP^+^/mCherry^+^ pixels) was calculated and compared. An increase in the number of colocalization events was visible over time (from 0% to 0.13% of events, Fig. S1b). This indicates that plasmid transmission occurs rapidly and can be monitored by the fluorescent system.

### Plasmid transmission is impacted by the ratio of donor and recipient bacteria.

**FIG 3:**
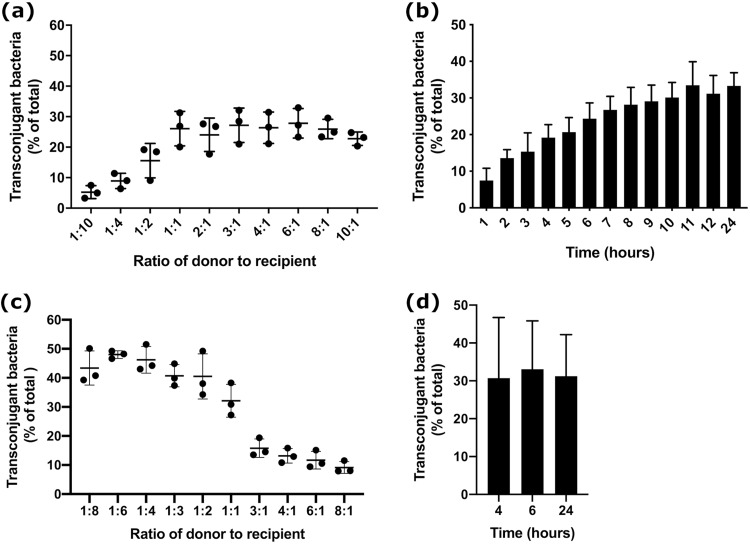
Measuring transmission using flow cytometry. (a) The impact of altering the ratio of donor to recipient E. coli on the amount of pCT*gfp* transconjugant bacteria (GFP^+^/mCherry^+^) after 24 h of coincubation. (b) E. coli pCT*gfp* transconjugants over time, mixed in a 3:1 donor/recipient ratio. (c) The impact of altering the ratio of donor to recipient K. pneumoniae on the amount of pKpQIL*gfp* transconjugant bacteria (GFP^+^/mCherry^+^) after 6 h of coincubation. (d) Transmission of pKpQIL*gfp* in K. pneumoniae at 4, 6, and 24 h after combination of donor and recipient in a 1:2 ratio. Data show the mean from three independent experiments composed of three biological replicates ± standard deviation.

The ratio of donor to recipient cells is known to impact the transfer efficiency of plasmids (57–59). Therefore, we used flow cytometry to measure the number of transconjugant/transconjugant daughter cells in a population after coincubation of donors and recipients at a range of donor-to-recipient ratios and time points. All bacteria were adjusted to an optical density of 0.5 at 600 nm (OD_600_) prior to conjugation experiments. For pCT*gfp* in E. coli, the highest number of transconjugants was observed after 24 h between ratios of 1:1 and 6:1 (Fig. 3a), with the number of transconjugants between 25 and 30% of cells. Based on these data, the optimal transmission ratio of 3 donor to 1 recipient was chosen for further experiments. To determine the optimal time point, transmission was measured every hour for 12 h and at 24 h using the 3:1 donor/recipient ratio. The level of transmission increased over time, with maximal numbers of transconjugants obtained between 11 and 24 h of coincubation (Fig. 3b).

For pKpQIL*gfp* transmission in K. pneumoniae, after 6 h the number of transconjugants peaked between the ratios of 1:8 and 1:2 (Fig. 3c). Ratios between 1:1 and 8:1 resulted in a sequential reduction in transconjugant populations (Fig. 3c). The ratio of 1:2 was selected for further experiments. The number of transconjugants produced was monitored at three time points: 4, 6, and 24 h. There was little change in the number of transconjugants between 4 and 24 h (Fig. 3d). Six hours was chosen for further experiments because at 24 h evidence of biofilm formation was observed, which is known to impact plasmid transmission (60–62).

### Effect of known plasmid-transmission inhibitors upon transmission of pCT and pKpQIL into E. coli and K. pneumoniae, respectively. (i) Chlorpromazine.

**FIG 4:**
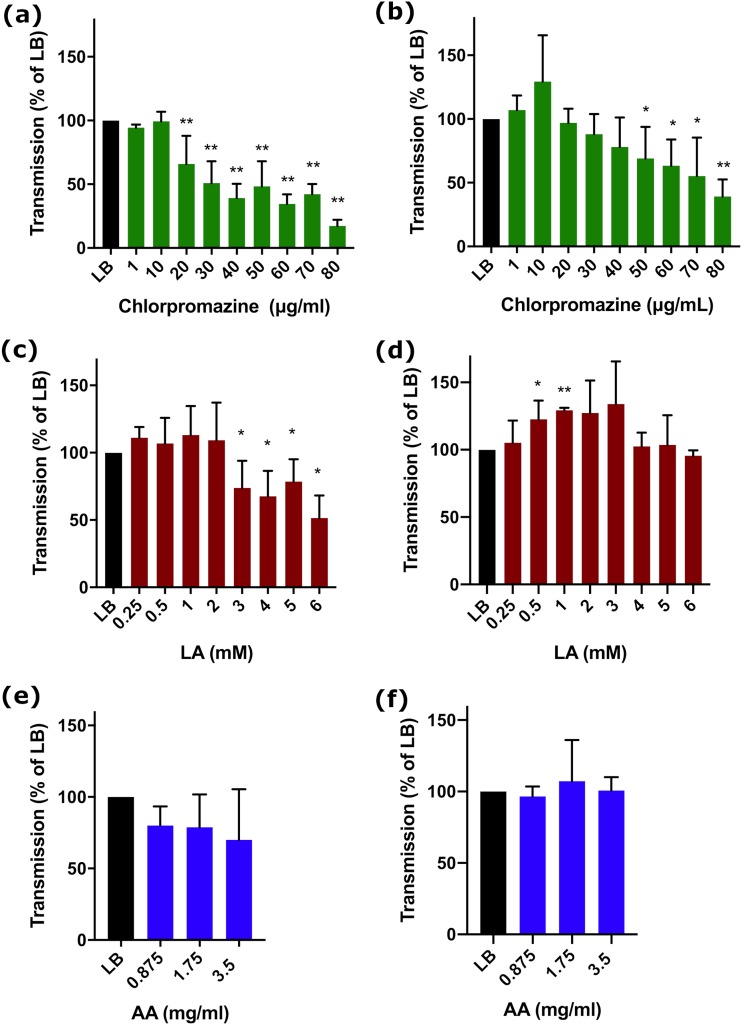
Dose response for the activity of antiplasmid compounds on plasmid transmission compared to LB alone. (a) The impact of chlorpromazine on pCT*gfp* transmission in E. coli after 24-h incubation. (b) The impact of chlorpromazine on pKpQIL*gfp* transmission in K. pneumoniae after 6-h incubation. (c) The impact of linoleic acid (LA) on pCT*gfp* transmission in E. coli after 24-h incubation. (d) The impact of linoleic acid on pKpQIL*gfp* transmission in K. pneumoniae after 6-h incubation. (e) The impact of ascorbic acid (AA) on pCT*gfp* transmission in E. coli after 24-h incubation. (d) The impact of ascorbic acid on pKpQIL*gfp* transmission in K. pneumoniae after 6-h incubation. Data show the mean ± standard deviation from a minimum of three independent experiments, each with a minimum of three biological replicates. *, *P* < 0.05; **, *P* < 0.001.

Chlorpromazine reduced transmission of both plasmids in both strains. For pCT*gfp* transmission in E. coli, after 24-h exposure concentrations of chlorpromazine of ≥20 μg/ml significantly reduced transmission (*P* < 0.001, Fig. 4a). The MIC of chlorpromazine was 128 μg/ml for all E. coli strains (Table 1). While the concentrations that inhibited transmission were below the MIC, we wanted to determine if chlorpromazine inhibited bacterial growth (as this may impact the composition of the population). Therefore, growth rate was determined in the presence of 30 μg/ml chlorpromazine. Some degree of growth inhibition was indicated by an increase in the generation time from 66 ± 8 min to 87 ± 10 min for ST131c, 75 ± 16 min to 96 ± 18 min for ST131 *mcherry*, and 77 ± 20 min to 93 ± 14 min for ST131c pCT*gfp*. Additional growth kinetic experiments revealed that a chlorpromazine concentration of 20 μg/ml had no impact upon growth rate of E. coli ST131 EC958 derivatives (Fig. S2a). Furthermore, 20 μg/ml chlorpromazine still reduced pCT*gfp* transmission (Fig. 4a).

**TABLE 1:** MICs of antibiotics and compounds

Strain	MIC of drug:								
	Kanamycin (μg/ml)^*a*^	Ampicillin (μg/ml)^*a*^	Cefotaxime (μg/ml)^*a*^	Nalidixic acid (μg/ml)^*b*^	Linoleic acid (mM)^*c*^	Ascorbic acid (mg/ml)^*c*^	Chlorpromazine (μg/ml)^*c*^	Abacavir (μg/ml)^*c*^	AZT (μg/ml)^*c*^
E. coli									
ST131	32	128	>256	>256	≥512	8	128	>512	ND^*d*^
ST131 pCure	4	<0.5	4	>256	ND	ND	ND	ND	ND
ST131c	4	<0.5	4	>256	≥512	8	128	>512	4
ST131c *mcherry*	ND	ND	ND	ND	≥512	8	128	>512	4
ST131c pCT*gfp*	ND	ND	ND	ND	≥512	8	128	>512	2
ST131 B104	4	ND	ND	ND	≥512	8	128	>512	ND
ST131 B104 *mcherry*	ND	ND	ND	ND	≥512	8	128	>512	ND
ST131 B104 pCT*gfp*	ND	ND	ND	ND	≥512	8	128	>512	ND
ST131c *mcherry* AZT-resistant mutant	ND	ND	ND	ND	ND	ND	ND	ND	>32
ST131c pCT*gfp* AZT-resistant mutant	ND	ND	ND	ND	ND	ND	ND	ND	>32
K. pneumoniae									
Ecl8	ND	ND	ND	2	>512	8	256	>512	2–4
Ecl8 pKpQIL*gfp*	ND	ND	ND	ND	>512	8	256	>512	2–4
Ecl8 *mcherry*	ND	ND	ND	ND	>512	4	256	>512	8–16
Ecl8 Nal^r^	ND	ND	ND	256	ND	ND	ND	ND	ND

a The resistance to these antibiotics is encoded on the pEC958 plasmid.

b The resistance to this antibiotic is encoded on the ST131 EC958 chromosome.

c Antiplasmid compounds.

d ND indicates that data were not required and so not determined.

10.1128/mBio.03355-19.3 FIG S2 Generation time of bacteria in LB alone or LB supplemented with either 3.5 mg/ml ascorbic acid (blue), 6 mM linoleic acid (red), 20 μg/ml chlorpromazine (green), or 8 μg/ml abacavir (purple). (a) E. coli ST131 strains. (b) K. pneumoniae Ecl8 strains. (c) ST131 B104 strains. (d) Dose-dependent effect of AZT on generation time of E. coli ST131c EC958 *mcherry* and pCT*gfp* strains and K. pneumoniae *mcherry* and pCT*gfp* strains. Data show the mean ± standard deviation from three independent experiments, comprised of at least three biological replicates. *, *P* < 0.05; **, *P* < 0.001; n.s., not significant. Download FIG S2, DOCX file, 2.3 MB. Copyright © 2020 Buckner et al. 2020 Buckner et al. This content is distributed under the terms of the Creative Commons Attribution 4.0 International license.

Concentrations of ≥50 μg/ml chlorpromazine resulted in a significant reduction in K. pneumoniae pKpQIL*gfp* transconjugants (Fig. 4b). The MIC of chlorpromazine for these strains was 256 μg/ml (Table 1). As with E. coli, 20 μg/ml chlorpromazine had no effect upon bacterial growth (Fig. S2b); however, this concentration did not reduce pKpQIL transconjugants. The concentration range that we tested goes above the peak serum concentrations of chlorpromazine (0.1 μg/ml) (63). We aimed to test concentrations of compounds that were within the therapeutic window. However, for some compounds such as chlorpromazine, only at higher concentrations were effects seen on bacterial plasmid transmission.

In order to validate the flow cytometry data, classical conjugation experiments with E. coli were performed. To enable these experiments, a rifampin-resistant recipient strain of ST131c was generated; PCR and sequencing indicated a mutation resulting in the substitution H537Y in the *rpoB* product. The MIC of rifampin for the wild type was 8 μg/ml and for the mutant was 1,024 μg/ml. The impact of chlorpromazine upon pCT*gfp* transmission was tested in liquid mating experiments, and the data obtained confirmed the results obtained by flow cytometry. In 20 μg/ml chlorpromazine, there was an 82% reduction in transconjugants compared to LB alone (Table 2). While the growth rate of the bacterial strains in 20 μg/ml chlorpromazine was unaffected, a 2.8-fold reduction in the viable counts of total bacteria after the classical conjugation assay was seen. This supported the hypothesis that chlorpromazine inhibits bacterial growth.

**TABLE 2:** Conjugation frequencies of pCT*gfp* from ST131 EC958 pCT*gfp* into ST131c in the presence of ascorbic acid (3.5 mg/ml), linoleic acid (6 mM), chlorpromazine (20 μg/ml), abacavir (8 μg/ml), or AZT (0.008 μg/ml)^*a*^

Compound	Conjugation frequency in compound (± SD)	Corresponding conjugation frequency in LB control (± SD)	% conjugation compared to LB control
Ascorbic acid	5.62 × 10^−8^ ± 9.47 × 10^−8^	1.15 × 10^−7^ ± 2.24 × 10^−7^	49
Linoleic acid	8.48 × 10^−10^ ± 3.28 × 10^−10^	1.15 × 10^−7^ ± 2.24 × 10^−7^	0.74
Chlorpromazine	4.24 × 10^−8^ ± 2.53 × 10^−8^	2.32 × 10^−7^ ± 1.74 × 10^−7^	18
Abacavir	6.22 × 10^−7^ ± 2.90 × 10^−7^	4.61 × 10^−7^ ± 2.55 × 10^−7^	135
AZT	2.34 × 10^−7^ ± 6.16 × 10^−8^	3.25 × 10^−7^ ± 1.39 × 10^−7^	72

a Data are the averages from three independent experiments, carried out with a minimum of three biological replicates each. Corresponding conjugation frequency in LB control represents frequency for the particular set of three independent experiments.

### (ii) Linoleic acid.

The unsaturated fatty acid linoleic acid was tested for inhibition of plasmid transmission. For E. coli, between 3 and 6 mM linoleic acid effectively reduced transmission of pCT*gfp* at 24 h (3 and 5 mM, *P* ≤ 0.05; 4 and 6 mM, *P* < 0.001) (Fig. 4c). The MIC of linoleic acid was ≥512 mM for all strains (Table 1); 6 mM linoleic acid had no effect upon growth of the ST131c strains (Fig. S2a). Estimates for the normal serum levels of linoleic acid in healthy individuals vary between studies but are in the range of 0.2 to 5.0 mM (64, 65). The classical conjugation assays confirmed a 99% reduction in transconjugants compared to LB alone (Table 2), without impacting the viable counts of either ST131c pCT*gfp* or ST131c *mcherry*.

For pKpQIL*gfp* transconjugants in K. pneumoniae, at 0.5 and 1 mM linoleic acid increased the number of transconjugants at 6 h (*P* = 0.047 and <0.001, respectively; Fig. 4d). No reduction in transconjugants was observed at any concentration tested. These concentrations are within the range which reduces transmission of other plasmids (48), including pCT (Fig. 4c), and are well below the MIC of linoleic acid (>512 mM, Table 1); 6 mM linoleic acid had no impact upon bacterial generation time (Fig. S2b).

### (iii) Ascorbic acid.

Ascorbic acid had no detectable impact on pCT*gfp* transmission in E. coli ST131c at any concentration when tested by flow cytometry up to the 24-h endpoint (Fig. 4e). However, ascorbic acid reduced the number of transconjugants when tested in a classical conjugation experiment (50% reduction [Table 2]). In order to identify antiplasmid activity of ascorbic acid, a concentration range at and above the upper peak serum concentration was used. The maximum concentration tested (3.5 mg/ml) was just below the MIC of ascorbic acid, 4 mg/ml (Table 1), and this concentration inhibited growth (increased the generation time of all strains [Fig. S2a]) and is above the peak serum concentration after intravenous (i.v.) treatment (2.4 mg/ml) (66). Ascorbic acid had no impact upon transmission of pKpQIL in K. pneumoniae (Fig. 4f), even at the high and growth-inhibiting concentration of 3.5 mg/ml; the MIC of ascorbic acid is 4 to 8 mg/ml (Table 1; Fig. S2b).

### An ESBL-free ST131 clinical isolate shows a similar pCT transmission profile as EC958.

**FIG 5:**
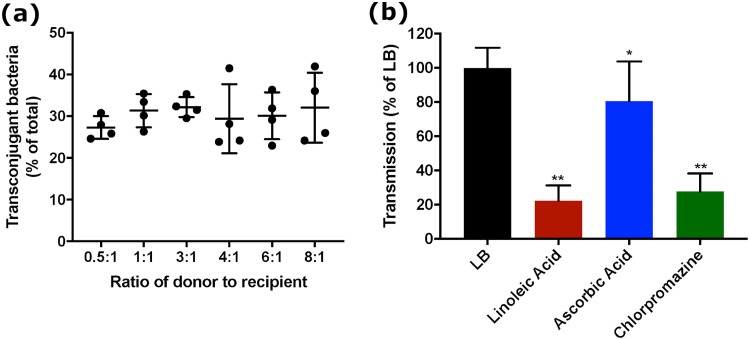
pCT*gfp* transmission dynamics in ST131 strain B104. (a) The impact of donor/recipient ratio on pCT*gfp* transmission. Each data point is the mean from four biological replicates; each data set comprises four independent experiments. (b) The impact of 6 mM linoleic acid, 3.5 mg/ml ascorbic acid, or 20 μg/ml chlorpromazine on pCT*gfp* transmission in B104 populations. Data show the mean ± standard deviation from four independent experiments, each with four biological replicates. *, *P* < 0.05; **, *P* < 0.001, calculated using Student’s *t* test.

To ensure that data obtained with E. coli ST131 EC958, which had been cured of its original plasmid, were not atypical, the effect of the antiplasmid compounds upon pCT transmission in a human clinical isolate of E. coli ST131 B104 with no identified plasmids (52) was measured. Both EC958 and B104 belong to ST131 clade C (52). For B104, a donor-to-recipient ratio of 3:1 resulted in the most consistent and highest average proportion of transconjugants (Fig. 5a). These data are similar to those for pCT*gfp* transmission in ST131c. This ratio was therefore used for further experiments. Linoleic acid (6 mM) and chlorpromazine (20 μg/ml) both significantly reduced the number of transmission events at 24 h (*P* < 0.001, Fig. 5b). The compounds had no effect on the growth of the strains (Fig. S2c). Overall, the data obtained were similar to those obtained for ST131c. Interestingly, ascorbic acid, which had no impact on pCT*gfp* transmission in ST131c, reduced transmission in B104 by 19% compared to LB alone (*P* < 0.05, Fig. 5b).

### Medium-throughput screening of the Prestwick library identified two FDA approved drugs with antiplasmid activity in both E. coli and K. pneumoniae.

The flow cytometry assay was used to screen the Prestwick Chemical Library comprising 1,280 molecules containing mostly approved drugs (FDA, European Medicines Agency [EMEA], and other agencies) selected for their high chemical and pharmacological diversity. This library was screened at 10 μM for compounds which reduce AMR plasmid transmission. From the screen results, all compounds known to be antibacterials or biocides were removed from the hit list, along with compounds which had a significant impact upon growth of bacteria (as measured by OD_600_ after incubation). These criteria reduced the number of hits to seven compounds: dexamethasone acetate, atropine sulfate monohydrate, abacavir sulfate, flavoxate hydrochloride, zidovudine (AZT), dolasetron mesylate, and pramipexole. Dexamethasone acetate was removed from the list as bacteria exposed to this compound produced very weak fluorescent signals. We decided to focus on two hits, abacavir and AZT, because they belong to the same class of drugs and they demonstrated promising results in three independent screening experiments for inhibition of transmission of both pCT and pKpQIL.

### (i) Abacavir.

**FIG 6:**
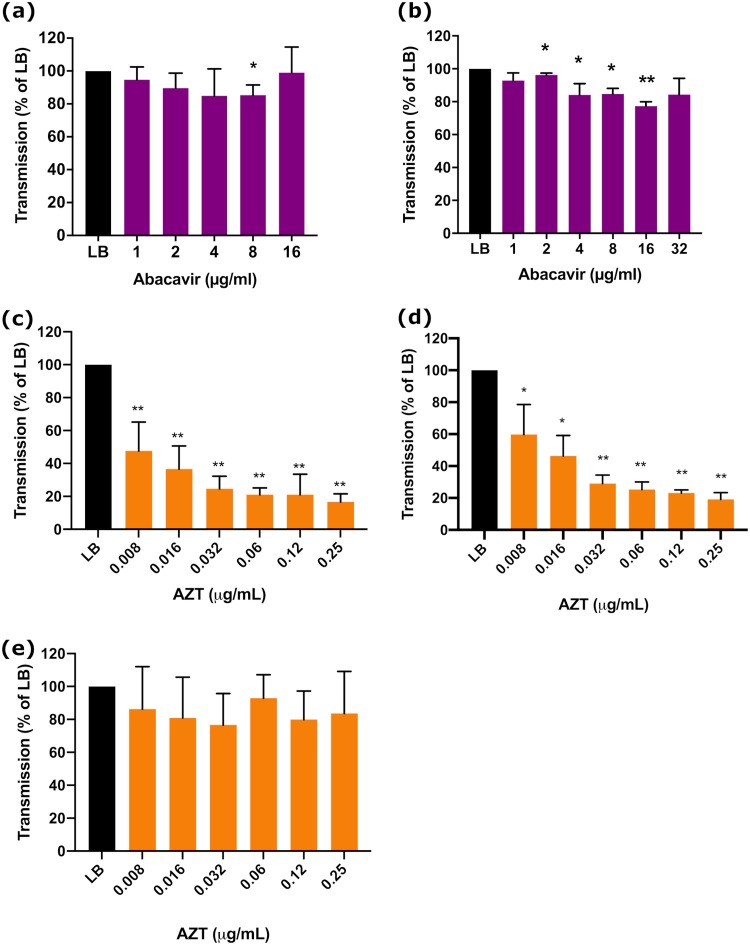
Abacavir and AZT impact AMR plasmid transmission. (a) pCT*gfp* transmission in ST131c EC958 E. coli after 24-h incubation with abacavir. (b) pKpQIL*gfp* transmission in K. pneumoniae after 6-h incubation with abacavir. (c) pCT*gfp* transmission in ST131c EC958 E. coli after 24-h incubation with AZT. (d) pKpQIL*gfp* transmission in K. pneumoniae after 6-h incubation with AZT. (e) AZT impact on transmission in AZT-resistant mutants of E. coli ST131c. ST131c pCT*gfp* and ST131c *mcherry* mutants were selected on 16 μg/ml AZT. Data show the mean ± standard deviation from a minimum of three independent experiments, each with at least three biological replicates. *, *P* < 0.05; **, *P* < 0.001, calculated using Student’s *t* test.

Abacavir did not inhibit growth of any bacterial strain (MIC of >512 μg/ml, Table 1), and 32 μg/ml of abacavir had no effect on the growth of any strain (Fig. S2a and b). To determine the optimal concentration for transmission inhibition, dose-response curves with abacavir were performed using flow cytometry. These demonstrated significant reduction of pCT*gfp* transmission in ST131c at 8 μg/ml abacavir (85.4% of the LB controls, *P* = 0.01) (Fig. 6a). Interestingly, 16 μg/ml had no impact on pCT*gfp* transmission (Fig. 6a). For E. coli ST131 B104, 16 μg/ml abacavir resulted in a significant reduction of pCT*gfp* transmission (85% of LB controls, *P* = 0.0036) (Fig. S3). Concentrations of >32 μg/ml abacavir affected recipient strain growth (data not shown). Classical conjugation assays showed that 8 μg/ml abacavir increased the number of transconjugants by 135% (Table 2). Abacavir is a well-characterized drug, and concentrations reached in the serum of patients being treated for HIV are typically 3 μg/ml (Table S1).

10.1128/mBio.03355-19.4 FIG S3 Impact of increasing concentrations of abacavir on pCT transmission in E. coli ST131 B104 strains. Data show mean ± standard deviation from three independent experiments, each with three biological replicates. *, *P* < 0.05; **, *P* < 0.001. Download FIG S3, DOCX file, 1.9 MB. Copyright © 2020 Buckner et al. 2020 Buckner et al. This content is distributed under the terms of the Creative Commons Attribution 4.0 International license.

10.1128/mBio.03355-19.5 TABLE S1 Characteristics of abacavir and zidovudine (AZT). Download Table S1, DOCX file, 0.03 MB. Copyright © 2020 Buckner et al. 2020 Buckner et al. This content is distributed under the terms of the Creative Commons Attribution 4.0 International license.

At 2 to 16 μg/ml, abacavir also reduced the number of pKpQIL*gfp* transconjugants; the largest reduction was seen at 16 μg/ml abacavir (77.4% of LB controls, *P* < 0.001) (Fig. 6b). To carry out a classical conjugation experiment, we generated a nalidixic acid-resistant mutant to act as the recipient strain; this allowed transconjugant bacteria to be distinguished by plating on a combination of kanamycin and nalidixic acid. Sequencing demonstrated an S83F mutation in *gyrA*, and the MIC of nalidixic acid for this strain was 256 μg/ml (Table 1). These mating experiments confirmed our flow cytometry data: 16 μg/ml abacavir resulted in a reduction in conjugation frequency to 63.0% ± 23.1% of LB controls (*P* = 0.0498; abacavir conjugation frequency, 1.58 × 10^−5^ ± 6.39 × 10^−6^; LB conjugation frequency, 2.88 × 10^−5^ ± 1.62 × 10^−5^).

### (ii) AZT.

AZT was also identified in our MTS experiments. Using flow cytometry, AZT caused a substantial, highly significant, and dose-dependent reduction in pCT transmission in E. coli (Fig. 6c); even the lowest concentration of AZT tested (0.008 μg/ml) resulted in a 52.4% reduction compared to LB alone (*P* = 0.0067); 0.25 μg/ml AZT reduced transconjugants by 83.3% ± 4.9% of LB controls (*P* < 0.0001). An AZT concentration of 0.008 μg/ml is between 1/512 and 1/256 the MIC of AZT for both strains (Table 1). As other studies have examined the antimicrobial activity of AZT (67–71), we also examined the impact of sub-MICs of AZT on bacterial growth. With increasing AZT concentrations, there was an increase in the generation time; at 0.008 μg/ml there was no effect on bacterial growth (Fig. S2d and e). This is well below peak serum concentrations, which range between 0.05 and 1.46 μg/ml (Table S1) (72). Together, this shows a concentration of AZT which reduces transmission of pCT by ∼50% and has no effect on bacterial growth. Supporting this, the classical conjugation experiments revealed that at 0.008 μg/ml, the conjugation frequency with AZT was 28% lower than the conjugation frequency in LB alone (Table 2).

There was a large reduction in the number of pKpQIL K. pneumoniae transconjugants formed in the presence of 0.008 to 0.25 μg/ml AZT (Fig. 6d); 0.25 μg/ml AZT reduced transconjugants by 80.8% ± 3.4% of LB controls (*P* < 0.0001). The MICs of AZT for the K. pneumoniae strains were 4 and 2 μg/ml for the Ecl8 parental strain and Ecl8 pKpQIL*gfp*, respectively, and the MIC was 16 μg/ml for Ecl8 *mcherry*. We consistently saw variation in the MIC between biological replicates within each experiment, with sporadic mutants appearing in wells with higher concentrations of AZT. In order to quantify this observation, we determined the mutation frequency of E. coli and K. pneumoniae strains to AZT (Table S2), which ranged from 8.65 × 10^−7^ to 1.45 × 10^−6^. This is in line with the observations of others (67, 68, 71, 73). Ecl8 *mcherry* grew more quickly at 0.016 and 0.032 μg/ml AZT, while above 0.12 μg/ml, AZT inhibited growth (Fig. S2d and e). Ecl8/pKpQIL*gfp* grew more slowly at ≥0.06 μg/ml AZT (Fig. S2d and e). At the lowest concentrations tested (0.008 to 0.032 μg/ml), there was no effect on growth of donors or recipients and these concentrations significantly reduced pKpQIL transmission (Fig. 6d).

10.1128/mBio.03355-19.6 TABLE S2 Mutation frequency of E. coli and K. pneumoniae strains to AZT. Download Table S2, DOCX file, 0.01 MB. Copyright © 2020 Buckner et al. 2020 Buckner et al. This content is distributed under the terms of the Creative Commons Attribution 4.0 International license.

AZT is a prodrug which is activated within the cell by phosphorylation events mediated by thymidine kinase to produce AZT-triphosphate, the active component in DNA chain termination (74). Single point mutations in thymidine kinase can result in AZT resistance (68, 73). Therefore, to determine if AZT-triphosphate was the active antiplasmid compound, we selected AZT-resistant E. coli mutants after exposure to 16 μg/ml AZT for 24 h. The mutation frequency to AZT (Table S2) was typical of that associated with a mutation in a single gene. The MIC of AZT for the mutants was >32 μg/ml. Sequencing of the thymidine kinase gene revealed a 10-bp deletion. The AZT-resistant mutants were then used in AZT transmission assays. AZT had no effect on plasmid transmission in the AZT-resistant mutants (Fig. 6e). This indicates that the active antiplasmid component is AZT-triphosphate.

## DISCUSSION

In this study, the dynamics of two globally disseminated, clinically relevant plasmids, producing either an ESBL or carbapenemase, were monitored for transmission in near real time into E. coli and K. pneumoniae populations, respectively. With fluorescently labeled plasmids and recipient bacteria, we monitored plasmid transmission among populations using flow cytometry.

We showed that chlorpromazine and linoleic acid function as inhibitors of an IncK CTX-M-producing plasmid transfer in E. coli and that chlorpromazine also functions as an antiplasmid compound for the IncFII KPC-producing plasmid pKpQIL in K. pneumoniae. Ascorbic acid had little/no effect on plasmid transmission, despite having a strong impact on bacterial growth. This shows that compounds that decrease growth do not necessarily also reduce plasmid transmission. While a variety of factors, including growth rate and low-level antibiotic selection, can impact the frequency of plasmid transfer (75, 76), we did not see such impact with ascorbic acid. Much of the work demonstrating the antiplasmid activity of ascorbic acid has been done with Gram-positive bacteria (45–47) and not Gram-negative bacteria as used here.

Chlorpromazine is a bioactive molecule which has many effects on the bacterial cell (63). These include intercalation into DNA molecules and possible breaks in single-stranded DNA (ssDNA) (77, 78). This intercalation could prevent the replication of the plasmid, resulting in plasmid loss from a population of bacteria. Chlorpromazine integrates into the lipid bilayer, impacting membrane permeability, fluidity, and some membrane-associated proteins (79–81). For example, chlorpromazine disrupted the activity of membrane-associated ATPases (82, 83). One can speculate that chlorpromazine may impact either the plasmid DNA itself, or the activity of the ATPases involved in the type IV secretion system used for conjugation, and thus inhibit plasmid transmission.

Here, we show that linoleic acid effectively inhibited conjugation of the IncK plasmid pCT at 24 h but had no impact on the IncFII plasmid pKpQIL in K. pneumoniae. Linoleic acid has previously been shown to inhibit conjugation of some plasmids, including IncF and IncW (48). Linoleic acid prevents conjugation by inhibiting the activity of the R388 plasmid TrwD ATPase, which is a type IV secretion traffic ATPase, and a homologue of Agrobacterium tumefaciens VirB11 (49, 84). The pCT plasmid encodes an ATPase in *trbB* (5), which in the plasmid RP4 encodes a homologue of VirB11 (85). Therefore, we hypothesize that the pCT TrbB is the target of linoleic acid-mediated transmission inhibition.

There was good correlation between the pCT flow cytometry data and the classical conjugation data. Both assays show that linoleic acid had the strongest antiplasmid activity, followed by chlorpromazine, AZT, and then abacavir. Interestingly, ascorbic acid reduced the conjugation frequency in classical conjugation experiments, but this was not detected in the ST131c flow cytometry experiments. It may be that the low level of activity of ascorbic acid is near the limit of detection of the flow cytometry assay. We hypothesize that any discrepancy between the conjugation assay and the flow cytometry assay may also relate to the large variation between conjugation assay experiments. Overall, the flow cytometry assay is optimal for medium-high-throughput experiments to identify compounds that inhibit plasmid transmission.

The flow cytometry assay was used to screen in medium throughput (hundreds of compounds/week) for compounds/drugs that inhibited transmission of pCT and pKpQIL within two species of bacteria. The two most promising hits from the Prestwick library were abacavir and AZT. While the impact of abacavir was modest, AZT had potent activity. These compounds have very different chemical structures from conjugation inhibitors such as linoleic acid and chlorpromazine. Abacavir and AZT are nucleoside analogue prodrugs that inhibit reverse transcriptase activity and are used clinically to treat HIV infection (see Table S1 in the supplemental material) (74, 86–88). AZT is converted into the active metabolite AZT-5′-triphosphate by thymidine kinases (67, 68), which is incorporated into DNA in place of thymidine, but since AZT lacks the 3′-hydroxy group, it results in chain termination (74). In the 1980s, AZT (2 μg/ml) was shown to inhibit the growth of E. coli and K. pneumoniae; however, AZT-resistant mutants with mutations in thymidine kinase were easily isolated *in vitro* (67); we made a similar observation in this study. Furthermore, AZT-resistant E. coli and *Salmonella* were recovered from patients receiving AZT treatment for HIV infection (89, 90). Recently, and in light of growing AMR, the antimicrobial properties of AZT have been explored (68–71, 73).

We hypothesize that the mechanism of antiplasmid activity of abacavir and AZT is that these compounds interfere with plasmid replication, including DNA chain termination, thus reducing plasmid transfer and/or plasmid presence in the population. This would explain our conjugation frequency data, which showed a smaller reduction in conjugation frequency in the presence of AZT than suggested by flow cytometry at the same concentration. Thus, when AZT reduces plasmid prevalence within the bacterial population, a substantial portion of cells may have lost the plasmid with the selectable marker (*aph*) used to distinguish donor/recipient/transconjugant bacteria; thus, in conjugation experiments such cells would skew the calculations used to determine conjugation frequency.

Two independent screens of FDA-approved compound libraries identified AZT as having activity against drug-resistant Gram-negative bacterial isolates (69, 70) and obtained MICs of AZT similar to those found in our study. The concentrations required to kill bacteria are within the peak serum concentrations for the drug (Table S1), and the use of already-approved drugs as new antibacterial compounds is attractive, as much of the pharmacokinetics (PK) and safety profiles are well established (74). In a study on human intestinal flora, 20 μM AZT affected growth of 10 bacteria including some strains of *Bacteroides*, *Bifidobacterium*, *Clostridium*, *Odoribacter*, *Roseburia*, and E. coli (91). Maier et al. (91) also estimated the intestinal concentrations of abacavir and AZT to be around 150 μM and 374 μM, respectively. These concentrations are much higher than the plasma concentrations of both drugs (Table S1) and, importantly, much higher than the concentrations which have antiplasmid properties. The concentration at which we saw antiplasmid activity (8 μg/ml abacavir corresponds to 27.9 μM; 0.008 μg/ml AZT corresponds to 0.029 μM) is within this range. Drug accumulation in the gut is ideal for an antiplasmid compound, as the intestinal flora is considered to be a hot spot for horizontal gene transfer, and dosage could be minimized, thus reducing potential systemic adverse effects. AZT treatment for HIV infection can result in AZT-resistant bacteria (89, 90). However, since the concentration which inhibits AMR plasmids is low (∼1/512 the MIC), the use of low doses as antiplasmid strategies would minimize the selection of resistant mutants. Furthermore, it is possible that although resistant bacteria could be selected at higher concentrations, such bacteria may no longer harbor plasmids carrying ARGs. To explore this hypothesis, clinical isolates of AZT-resistant bacteria should be examined for the presence of plasmids.

AMR plasmids are frequently found in the normal bacterial flora, in particular in the gastrointestinal (GI) tract (10, 11, 18–20, 41, 42). While it is clear that AMR plasmids are found and can persist within the GI tract, details of the intricacies of the AMR plasmid-microbiome relationships are still unclear. The impact of antiplasmid compounds on the AMR plasmid-microbiome relationship, including the impact of plasmid copy number and evolution, remains to be elucidated. However, we anticipate that improved models of AMR plasmid dynamics in the microbiome will allow the opportunity to address the impact of these factors on antiplasmid compound efficacy. In future, the testing of compounds such as abacavir and AZT in multispecies experiments will provide important insight into potential pathogen-microbiome interactions.

Before antiplasmid compounds can be used to reduce AMR prevalence in bacterial populations, additional factors should be considered. These include the potential differential activities of compounds on different plasmids and hosts. As shown here, compound activity may not be ubiquitous against all plasmids/bacteria. Furthermore, clinical strains can contain multiple plasmids; the impact of compounds in these settings should be examined. In complex, multispecies environments, plasmid-containing strains may also form the minority within the population; therefore, impact of compounds on minority populations and plasmid-free cells should be considered.

Additional chemical modifications and/or homologues may improve the antiplasmid activity and/or reduce antibacterial properties of abacavir and AZT, thus improving activity and alleviating any potential for selection pressure for AZT resistance. The use of nontoxic compounds to limit the spread of AMR plasmids (as opposed to killing the bacteria and thus reducing selective pressure) in key areas where transmissible ARGs are prevalent could be an effective means of reducing critical AMR in bacteria.

Identification of antiplasmid compounds has been slow, in large part due to dependence upon laborious plating experiments, which are not amenable to medium-/high-throughput screening. Apart from one screening system for conjugation inhibitors (48, 92, 93), most studies of antiplasmid compounds have used classical conjugation experiments. We developed and applied a fluorescence-based assay to monitor the movement of globally prevalent plasmids associated with human infections in two different Gram-negative species with plasmids carrying ESBL or carbapenemase genes. Furthermore, we used this assay in a medium-throughput screen and identified novel antiplasmid compounds. We uncovered the antiplasmid properties of two currently licensed and widely used anti-HIV drugs, abacavir and AZT. In light of the challenges associated with developing new antibiotics, nucleoside analogue drugs could be used to reduce the prevalence of drug-resistant Gram-negative bacteria via decolonization of vulnerable patients so that if they succumb to an infection a currently licensed drug will be effective.

## MATERIALS AND METHODS

### Bacterial strains, growth, and measurement of drug susceptibility.

The ST131 strain EC958 was isolated from a patient with a community-acquired UTI in Northwest England and has been used as a representative of this clonal group (50, 51). It is a multidrug-resistant (MDR) strain, carrying a pEK499-like 136-kb plasmid, pEC958 (50, 51). pEC958 is an IncF plasmid with two replicons, RepFII and RepFIA, and 12 resistance genes, including CTX-M-15 (55). The pKpQIL plasmid was used as previously described (termed pKpQIL-UK in publications [38, 94]). Repeated attempts to establish the fluorescent system in K. pneumoniae from the ST258 lineage were unsuccessful. Therefore, we used K. pneumoniae Ecl8, which has been used previously (38, 53, 54) by others and ourselves for successful cloning applications. Details regarding sequencing, culture conditions, growth kinetics assays, MIC assays, and pEC958 curing can be found in Text S1 and the list of plasmids, strains, and primers can be found in Table S3, both in the supplemental material.

10.1128/mBio.03355-19.7 TABLE S3 Bacterial strains, plasmids, and primers used in this study. Download Table S3, DOCX file, 0.03 MB. Copyright © 2020 Buckner et al. 2020 Buckner et al. This content is distributed under the terms of the Creative Commons Attribution 4.0 International license.

10.1128/mBio.03355-19.8 TABLE S4 Single nucleotide polymorphisms (SNPs) present in pEC958 plasmid in ST131 EC958 compared to reference pEC958 (HG941719). Download Table S4, XLSX file, 0.01 MB. Copyright © 2020 Buckner et al. 2020 Buckner et al. This content is distributed under the terms of the Creative Commons Attribution 4.0 International license.

### Construction of *gfp* reporters.

pCT_CTX-M-14_ was previously modified by insertion of *gfp-aph* under the control of the *acpP* promoter into *bla*_CTX-M_ and stored in E. coli DH5α (95). Filter matings (per reference 38) were used to move pCT*gfp* from DH5α into ST131c, the variant of ST131 EC958 cured using an incompatibility system (courtesy of Christopher M. Thomas), and transconjugants were selected on 256 μg/ml nalidixic acid and 75 μg/ml kanamycin. Plasmid presence was confirmed by PCR, and host strain identity was confirmed by testing ciprofloxacin resistance, confirming previously reported MIC values (55), and whole-genome sequencing (WGS; details available in Text S1). Expression of *gfp* was confirmed by flow cytometry, as described below.

For pKpQIL*gfp* construction, pUA66 was used as the template, and PCR was used to amplify *gfp-mut2* under the control of the *acpP* promoter with *aph* located downstream of *gfp-mut2*. E. coli SW105 containing temperature-sensitive recombinase and pKpQIL was made electrocompetent, and *gfp-mut2* PCR product was transformed into the cells. Successful insertions were selected on kanamycin (50 μg/ml) and confirmed by PCR and sequencing. The constructed plasmid pKpQIL*gfp* was transformed into E. coli DH10B for long-term storage. K. pneumoniae Ecl8 was made electrocompetent, and pKpQIL*gfp* was transformed into cells, selected for using 50 μg/ml kanamycin, and confirmed by PCR and sequencing. Expression of GFP was confirmed by flow cytometry.

### Construction of mCherry-expressing recipient strains.

To prepare the *mcherry* fragment, the *acpP* promoter was amplified from pUA66 (L1019), using primers with HindIII and BamHI sites; this was then digested and inserted into *mcherry* encoding pET-17b. This resulted in pET-17b p*acpP-mcherry*. The *aph* cassette was amplified from pKD4, with primers inserting XhoI sites up- and downstream of the gene. Restriction digestion and ligation were carried out to insert *aph* downstream of *mcherry.* PCR was used to confirm the direction of the *aph* cassette, and the final construct (pET-17b p*acpP-mcherry-aph*) was confirmed by PCR and sequencing. PCR was used to amplify the p*acpP-mcherry-aph* region, with primers containing a 45-bp overlap corresponding to the *putPA* intergenic region in E. coli or K. pneumoniae. *putPA* was chosen because this locus has been previously shown to minimize impact and allow expression of fluorescent proteins (56).

The pSIM18 plasmid, expressing recombinase machinery under a temperature-sensitive promoter (96), was transformed into electrocompetent E. coli or K. pneumoniae. pSIM18-containing colonies were selected using 150 μg/ml hygromycin at 30°C. Expression of the recombinase machinery was induced, and pSIM18 cells were made electrocompetent; the p*acpP-mcherry-aph* PCR product was transformed into these cells. After recovery at 37°C, cells were subcultured on plates containing 50 μg/ml kanamycin. Successful insertion events were identified by PCR amplification of the inserted region, and loss of hygromycin resistance (pSIM18) was tested. Expression of *mcherry* was confirmed by flow cytometry, as described below.

### Construction of fluorescent reporters in E. coli ST131 B104.

A naturally plasmid-free E. coli ST131 clinical isolate, B104 (52), was modified for use in the pCT transmission assay. First, to construct the B104 donor strain, a rifampin-resistant B104 strain was generated by overnight growth on LB agar containing 100 μg/ml rifampin. Candidate colonies were selected, and growth kinetics were determined to ensure that mutation did not impact growth rate. Sequencing of the *rpoB* gene was performed to identify the mutations resulting in rifampin resistance. Then, conjugation was used to move pCT*gfp* from ST131c pCT*gfp* into B104^RifR^, resulting in the construction of B104 pCT*gfp*. PCR was used to confirm the presence of pCT*gfp*, and flow cytometry was used to check fluorescence.

To construct the B104 recipient strain, the recombineering plasmid pSLTS (97) was first transformed into B104 by electroporation, as described above. PCR was used to amplify the p*acpP-mcherry-aph* region from pET-17b p*acpP-mcherry-aph*, with primers containing a 45-bp overlap corresponding to the *putPA* intergenic region in B104. This product was transformed into electrocompetent B104 pSLTS, similar to what is described above and according to reference 97. Successful recombination events were selected on 50 μg/ml kanamycin. PCR was used to confirm insertion of *mcherry*, and flow cytometry was used to check fluorescence.

### Transmission assay.

Overnight cultures of donor (E. coli with pCT*gfp* or K. pneumoniae with pKpQIL*gfp*) and recipients (E. coli or K. pneumoniae with chromosomal *mcherry*) were washed in phosphate-buffered saline (PBS) (Sigma) and diluted to an optical density (600 nm) of 0.5. Bacteria were combined at a donor/recipient ratio of 3:1 for E. coli and 1:2 for K. pneumoniae, unless otherwise specified. This mixture (20 μl) was used to inoculate 180 μl of LB broth in a 96-well plate. Controls included LB broth alone, donor alone, and recipient alone. The plate was incubated at 37°C with gentle agitation (∼80 rpm) for the specified amount of time. At each time point, 20 μl was removed and serially diluted to 1:1,000 in PBS filtered through 0.2-μm filters. Samples were run on the Attune NxT acoustic focusing flow cytometer with Autosampler (Thermo Scientific), equipped with a blue/yellow (excitation laser: blue, 488 nm; yellow, 561 nm) laser configuration; additional details can be found in Text S1. Flow cytometry has been previously used to monitor plasmids within populations (for examples, see references 98 and 99).

Ascorbic acid, chlorpromazine, linoleic acid, abacavir, and AZT were added to LB in 96-well plates, over a range of concentrations. Ascorbic acid stock solutions of 100 mg/ml were prepared in sterile distilled water, chlorpromazine stock solutions of 10,000 μg/ml were prepared in water, linoleic acid stock solutions of 100 mM were prepared in 30% dimethyl sulfoxide (DMSO), abacavir sulfate stock solutions of 10,000 μg/ml were prepared in water, and AZT stock solutions of 1,000 μg/ml were prepared in methanol. All transmission assays were completed with a minimum of three independent experiments, with between three and four biological replicates per experiment.

For pCT*gfp* transmission in B104 strains, transmission assays were set up exactly as described above, with different donor/recipient ratios tested. Antiplasmid compounds were tested at the following concentrations: 3.5 mg/ml ascorbic acid, 6 mM linoleic acid, and 20 μg/ml chlorpromazine.

Details of how conjugation frequencies were determined can be found in Text S1.

### Determining frequency of mutation for AZT resistance and selection of AZT-resistant mutants.

To determine AZT frequency of mutation, ST131c, ST131 pCT*gfp*, and ST131 *mcherry* were plated onto LB agar plates containing 16 μg/ml AZT and LB agar plates containing no drug. Plates were incubated for 24 h at 37°C, and the colonies were enumerated. The Don Whitley Scientific automated spiral plater was used, per the manufacturer’s instructions. For each strain, 4 biological and 5 technical replicates were used. Mutation frequency was calculated by dividing the number of mutants by the viable count. From the AZT plates, mutant colonies were selected, and the MIC of AZT was determined as indicated above. Candidates with an AZT MIC of >32 μg/ml were selected, and the thymidine kinase gene was sequenced to determine if mutations had occurred (primers listed in Table S3). Mutants with deletions in thymidine kinase were then used in the AZT flow cytometry assay.

### Fluorescent microscopy.

Multichannel confocal images were taken at the Birmingham Light Advance Microscopy (BALM) facility at the University of Birmingham. In order to allow a proper comparison between different samples, the same optical configuration (digital zoom, photomultiplier tube [PMT], laser power, Galvano scanner, and pinhole) was used in all acquisitions. Samples were mixed to a final volume of 6 μl in morpholinepropanesulfonic acid (MOPS) minimal medium alone or with ascorbic acid (3.5 mg/ml), linoleic acid (6 μM), or chlorpromazine (50 μg/ml). A higher chlorpromazine concentration was used because of the short duration of this experiment. Bacteria were mounted on a conventional glass slide, placed on a heated stage (37°C), and immediately imaged every 10 min over 120 min. Time-lapse acquisition was performed sequentially by using the 488-nm and the 543-nm laser lines on a Zeiss LSM710 Confocor3 point scanning confocal system mounted on an Axio Observer inverted microscope with a 63×/1.4 oil objective (Zeiss).

Image analysis was performed on the open-source software ICY (http://icy.bioimageanalysis.org/) using the Wavelet Spot Detector and the Colocalization Studio plugins to assess the level of colocalization between the two different cells over time. A total of 12 images across 3 replicates for each time point and condition were analyzed. The number of colocalization events was normalized to the total number of bacteria and plotted using GraphPad Prism.

### High-throughput screening.

The Prestwick Chemical Library, containing 1,280 drugs and compounds, was tested in 96-well format using a Hamilton Star robot to identify compounds which inhibited the transmission of pCT*gfp* in ST131c or pKpQIL in *K. pneumoniae*. Compounds were dissolved in DMSO. A final concentration of 10 μM compound was added to the assay plate containing LB broth and a 3:1 or 1:2 mixture of donor and recipient cells (as described above). The final concentration of DMSO was tested and had no impact on bacterial growth or plasmid transmission. Plates were incubated for 24 h and read on the Attune flow cytometer as described above. The OD_600_ of each plate was determined using a FLUOstar Optima, as described above. Each compound plate was repeated in two independent experiments. Plates containing promising compounds were repeated a third time. The number of transconjugants in each well was determined and compared with LB controls. Compounds with levels of transmission below LB values were considered possible hits.

10.1128/mBio.03355-19.9 TABLE S5 SNPs present in all four strains (ST131, ST131c, ST131c pCT*gfp*, and ST131c *mcherry*) compared to EC958 reference sequence (NZ_HG941718) and SNPs located in some, but not all, of the four strains, as indicated. Download Table S5, XLSX file, 0.02 MB. Copyright © 2020 Buckner et al. 2020 Buckner et al. This content is distributed under the terms of the Creative Commons Attribution 4.0 International license.

10.1128/mBio.03355-19.10 TABLE S6 SNPs present in pCT*gfp* compared with the pCT reference sequence (NC_014477.1). Download Table S6, XLSX file, 0.01 MB. Copyright © 2020 Buckner et al. 2020 Buckner et al. This content is distributed under the terms of the Creative Commons Attribution 4.0 International license.

## References

[1] WHO. 2017 Global priority list of antibiotic-resistant bacteria to guide research, discovery, and development of new antibiotics. WHO, Geneva, Switzerland.

[2] O’Neill J 2016 Tackling drug-resistant infections globally: final report and recommendations. Review on Antimicrobial Resistance, London, United Kingdom.

[3] CDC. 2013 Antibiotic resistance threats in the United States. CDC, Atlanta, GA.

[4] Liu Y-Y, Wang Y, Walsh TR, Yi L-X, Zhang R, Spencer J, Doi Y, Tian G, Dong B, Huang X, Yu L-F, Gu D, Ren H, Chen X, Lv L, He D, Zhou H, Liang Z, Liu J-H, Shen J 2016 Emergence of plasmid-mediated colistin resistance mechanism MCR-1 in animals and human beings in China: a microbiological and molecular biological study. Lancet Infect Dis 16:161–168. doi:10.1016/S1473-3099(15)00424-7. 26603172

[5] Cottell JL, Webber MA, Coldham NG, Taylor DL, Cerdeño-Tárraga AM, Hauser H, Thomson NR, Woodward MJ, Piddock LV 2011 Complete sequence and molecular epidemiology of IncK epidemic plasmid encoding *bla*(CTX-M-14). Emerg Infect Dis 17:645–652. doi:10.3201/eid1704.101009. 21470454 PMC3377399

[6] Doumith M, Findlay J, Hirani H, Hopkins KL, Livermore DM, Dodgson A, Woodford N 2017 Major role of pKpQIL-like plasmids in the early dissemination of KPC-type carbapenemases in the UK. J Antimicrob Chemother 72:2241–2248. doi:10.1093/jac/dkx141. 28498924

[7] Tacconelli E, WHO Pathogens Priority List Working Group, Carrara E, Savoldi A, Harbarth S, Mendelson M, Monnet DL, Pulcini C, Kahlmeter G, Kluytmans J, Carmeli Y, Ouellette M, Outterson K, Patel J, Cavaleri M, Cox EM, Houchens CR, Grayson ML, Hansen P, Singh N, Theuretzbacher U, Magrini N 2018 Discovery, research, and development of new antibiotics: the WHO priority list of antibiotic-resistant bacteria and tuberculosis. Lancet Infect Dis 18:318–327. doi:10.1016/S1473-3099(17)30753-3. 29276051

[8] CDC. 2019 Biggest threats and data. 2019 AR threats report. CDC, Atlanta, GA.

[9] Stecher B, Denzler R, Maier L, Bernet F, Sanders MJ, Pickard DJ, Barthel M, Westendorf AM, Krogfelt KA, Walker AW, Ackermann M, Dobrindt U, Thomson NR, Hardt W-D 2012 Gut inflammation can boost horizontal gene transfer between pathogenic and commensal *Enterobacteriaceae*. Proc Natl Acad Sci U S A 109:1269–1274. doi:10.1073/pnas.1113246109. 22232693 PMC3268327

[10] Gumpert H, Kubicek-Sutherland JZ, Porse A, Karami N, Munck C, Linkevicius M, Adlerberth I, Wold AE, Andersson DI, Sommer M 2017 Transfer and persistence of a multi-drug resistance plasmid in situ of the infant gut microbiota in the absence of antibiotic treatment. Front Microbiol 8:1852. doi:10.3389/fmicb.2017.01852. 29018426 PMC5622998

[11] Jorgensen TS, Xu Z, Hansen MA, Sorensen SJ, Hansen LH 2014 Hundreds of circular novel plasmids and DNA elements identified in a rat cecum metamobilome. PLoS One 9:e87924. doi:10.1371/journal.pone.0087924. 24503942 PMC3913684

[12] Amos GCA, Hawkey PM, Gaze WH, Wellington EM 2014 Waste water effluent contributes to the dissemination of CTX-M-15 in the natural environment. J Antimicrob Chemother 69:1785–1791. doi:10.1093/jac/dku079. 24797064 PMC4054988

[13] Rahube TO, Marti R, Scott A, Tien Y-C, Murray R, Sabourin L, Duenk P, Lapen DR, Topp E 2016 Persistence of antibiotic resistance and plasmid-associated genes in soil following application of sewage sludge and abundance on vegetables at harvest. Can J Microbiol 62:600–607. doi:10.1139/cjm-2016-0034. 27277701

[14] Klumper U, Riber L, Dechesne A, Sannazzarro A, Hansen LH, Sorensen SJ, Smets BF 2015 Broad host range plasmids can invade an unexpectedly diverse fraction of a soil bacterial community. ISME J 9:934–945. doi:10.1038/ismej.2014.191. 25333461 PMC4817699

[15] Klumper U, Dechesne A, Riber L, Brandt KK, Gulay A, Sorensen SJ, Smets BF 2017 Metal stressors consistently modulate bacterial conjugal plasmid uptake potential in a phylogenetically conserved manner. ISME J 11:152–165. doi:10.1038/ismej.2016.98. 27482924 PMC5097465

[16] Kotay S, Chai W, Guilford W, Barry K, Mathers AJ 2017 Spread from the sink to the patient: in situ study using green fluorescent protein (GFP)-expressing *Escherichia coli* to model bacterial dispersion from hand-washing sink-trap reservoirs. Appl Environ Microbiol 83:e03327-16. doi:10.1128/AEM.03327-16. 28235877 PMC5377511

[17] Kizny Gordon AE, Mathers AJ, Cheong EYL, Gottlieb T, Kotay S, Walker AS, Peto TEA, Crook DW, Stoesser N 2017 The hospital water environment as a reservoir for carbapenem-resistant organisms causing hospital-acquired infections—a systematic review of the literature. Clin Infect Dis 64:1435–1444. doi:10.1093/cid/cix132. 28200000

[18] Arcilla MS, van Hattem JM, Haverkate MR, Bootsma MCJ, van Genderen PJJ, Goorhuis A, Grobusch MP, Lashof AMO, Molhoek N, Schultsz C, Stobberingh EE, Verbrugh HA, de Jong MD, Melles DC, Penders J 2017 Import and spread of extended-spectrum β-lactamase-producing *Enterobacteriaceae* by international travellers (COMBAT study): a prospective, multicentre cohort study. Lancet Infect Dis 17:78–85. doi:10.1016/S1473-3099(16)30319-X. 27751772

[19] Vading M, Kabir MH, Kalin M, Iversen A, Wiklund S, Nauclér P, Giske CG 2016 Frequent acquisition of low-virulence strains of ESBL-producing *Escherichia coli* in travellers. J Antimicrob Chemother 71:3548–3555. doi:10.1093/jac/dkw335. 27566312

[20] Pires J, Kuenzli E, Kasraian S, Tinguely R, Furrer H, Hilty M, Hatz C, Endimiani A 2016 Polyclonal intestinal colonization with extended-spectrum cephalosporin-resistant Enterobacteriaceae upon traveling to India. Front Microbiol 7:1069. doi:10.3389/fmicb.2016.01069. 27462305 PMC4940376

[21] Stokes MO, Cottell JL, Piddock LV, Wu G, Wootton M, Mevius DJ, Randall LP, Teale CJ, Fielder MD, Coldham NG 2012 Detection and characterization of pCT-like plasmid vectors for blaCTX-M-14 in *Escherichia coli* isolates from humans, turkeys and cattle in England and Wales. J Antimicrob Chemother 67:1639–1644. doi:10.1093/jac/dks126. 22514265

[22] Rozwandowicz M, Brouwer MSM, Zomer AL, Bossers A, Harders F, Mevius DJ, Wagenaar JA, Hordijk J 2017 Plasmids of distinct IncK lineages show compatible phenotypes. Antimicrob Agents Chemother 61:e01954-16. doi:10.1128/AAC.01954-16. 28052854 PMC5328535

[23] Bevan ER, Jones AM, Hawkey PM 2017 Global epidemiology of CTX-M beta-lactamases: temporal and geographical shifts in genotype. J Antimicrob Chemother 72:2145–2155. doi:10.1093/jac/dkx146. 28541467

[24] Valverde A, Cantón R, Garcillán-Barcia MP, Novais Â, Galán JC, Alvarado A, de la Cruz F, Baquero F, Coque TM 2009 Spread of *bla*CTX-M-14 is driven mainly by IncK plasmids disseminated among Escherichia coli phylogroups A, B1, and D in Spain. Antimicrob Agents Chemother 53:5204–5212. doi:10.1128/AAC.01706-08. 19786598 PMC2786348

[25] Dhanji H, Khan P, Cottell JL, Piddock LV, Zhang J, Livermore DM, Woodford N 2012 Dissemination of pCT-like IncK plasmids harboring CTX-M-14 extended-spectrum β-lactamase among clinical Escherichia coli isolates in the United Kingdom. Antimicrob Agents Chemother 56:3376–3377. doi:10.1128/AAC.00313-12. 22450980 PMC3370816

[26] Cottell JL, Saw HTH, Webber MA, Piddock LV 2014 Functional genomics to identify the factors contributing to successful persistence and global spread of an antibiotic resistance plasmid. BMC Microbiol 14:168. doi:10.1186/1471-2180-14-168. 24961279 PMC4083329

[27] Cottell JL, Webber MA, Piddock LV 2012 Persistence of transferable extended-spectrum-β-lactamase resistance in the absence of antibiotic pressure. Antimicrob Agents Chemother 56:4703–4706. doi:10.1128/AAC.00848-12. 22710119 PMC3421869

[28] Stoesser N, Modernizing Medical Microbiology Informatics Group (MMMIG), Sheppard AE, Pankhurst L, De Maio N, Moore CE, Sebra R, Turner P, Anson LW, Kasarskis A, Batty EM, Kos V, Wilson DJ, Phetsouvanh R, Wyllie D, Sokurenko E, Manges AR, Johnson TJ, Price LB, Peto TEA, Johnson JR, Didelot X, Walker AS, Crook DW 2016 Evolutionary history of the global emergence of the *Escherichia coli* epidemic clone ST131. mBio 7:e02162-15. doi:10.1128/mBio.02162-15. 27006459 PMC4807372

[29] Croxall G, Hale J, Weston V, Manning G, Cheetham P, Achtman M, McNally A 2011 Molecular epidemiology of extraintestinal pathogenic *Escherichia coli* isolates from a regional cohort of elderly patients highlights the prevalence of ST131 strains with increased antimicrobial resistance in both community and hospital care setting. J Antimicrob Chemother 66:2501–2508. doi:10.1093/jac/dkr349. 21862477

[30] Leavitt A, Chmelnitsky I, Carmeli Y, Navon-Venezia S 2010 Complete nucleotide sequence of KPC-3-encoding plasmid pKpQIL in the epidemic Klebsiella pneumoniae sequence type 258. Antimicrob Agents Chemother 54:4493–4496. doi:10.1128/AAC.00175-10. 20696875 PMC2944570

[31] Eilertson B, Chen L, Chavda KD, Kreiswirth BN 2017 Genomic characterization of two KPC-producing *Klebsiella* isolates collected in 1997 in New York City. Antimicrob Agents Chemother 61:e02458-16. doi:10.1128/AAC.02458-16. 28167551 PMC5365676

[32] Chen L, Mathema B, Chavda KD, DeLeo FR, Bonomo RA, Kreiswirth BN 2014 Carbapenemase-producing *Klebsiella pneumoniae*: molecular and genetic decoding. Trends Microbiol 22:686–696. doi:10.1016/j.tim.2014.09.003. 25304194 PMC4365952

[33] Baraniak A, KPC-PL Study Group, Grabowska A, Izdebski R, Fiett J, Herda M, Bojarska K, Żabicka D, Kania-Pudło M, Młynarczyk G, Żak-Puławska Z, Hryniewicz W, Gniadkowski M 2011 Molecular characteristics of KPC-producing *Enterobacteriaceae* at the early stage of their dissemination in Poland, 2008–2009. Antimicrob Agents Chemother 55:5493–5499. doi:10.1128/AAC.05118-11. 21930889 PMC3232751

[34] Woodford N, Zhang J, Warner M, Kaufmann ME, Matos J, Macdonald A, Brudney D, Sompolinsky D, Navon-Venezia S, Livermore DM 2008 Arrival of *Klebsiella pneumoniae* producing KPC carbapenemase in the United Kingdom. J Antimicrob Chemother 62:1261–1264. doi:10.1093/jac/dkn396. 18812425

[35] Findlay J, Hopkins KL, Doumith M, Meunier D, Wiuff C, Hill R, Pike R, Loy R, Mustafa N, Livermore DM, Woodford N 2016 KPC enzymes in the UK: an analysis of the first 160 cases outside the North-West region. J Antimicrob Chemother 71:1199–1206. doi:10.1093/jac/dkv476. 26846210 PMC4830413

[36] Grundmann H, European Survey of Carbapenemase-Producing Enterobacteriaceae (EuSCAPE) Working Group, Glasner C, Albiger B, Aanensen DM, Tomlinson CT, Andrasević AT, Cantón R, Carmeli Y, Friedrich AW, Giske CG, Glupczynski Y, Gniadkowski M, Livermore DM, Nordmann P, Poirel L, Rossolini GM, Seifert H, Vatopoulos A, Walsh T, Woodford N, Monnet DL 2017 Occurrence of carbapenemase-producing *Klebsiella pneumoniae* and *Escherichia coli* in the European survey of carbapenemase-producing Enterobacteriaceae (EuSCAPE): a prospective, multinational study. Lancet Infect Dis 17:153–163. doi:10.1016/S1473-3099(16)30257-2. 27866944

[37] Martin J, Phan HTT, Findlay J, Stoesser N, Pankhurst L, Navickaite I, De Maio N, Eyre DW, Toogood G, Orsi NM, Kirby A, Young N, Turton JF, Hill RLR, Hopkins KL, Woodford N, Peto TEA, Walker AS, Crook DW, Wilcox MH 2017 Covert dissemination of carbapenemase-producing Klebsiella pneumoniae (KPC) in a successfully controlled outbreak: long- and short-read whole-genome sequencing demonstrate multiple genetic modes of transmission. J Antimicrob Chemother 72:3025–3034. doi:10.1093/jac/dkx264. 28961793 PMC5890743

[38] Buckner MMC, Saw HTH, Osagie RN, McNally A, Ricci V, Wand ME, Woodford N, Ivens A, Webber MA, Piddock L 2018 Clinically relevant plasmid-host interactions indicate that transcriptional and not genomic modifications ameliorate fitness costs of *Klebsiella pneumoniae* carbapenemase-carrying plasmids. mBio 9:e02303-17. doi:10.1128/mBio.02303-17. PMC5915730 29691332

[39] Leavitt A, Navon-Venezia S, Chmelnitsky I, Schwaber MJ, Carmeli Y 2007 Emergence of KPC-2 and KPC-3 in carbapenem-resistant Klebsiella pneumoniae strains in an Israeli hospital. Antimicrob Agents Chemother 51:3026–3029. doi:10.1128/AAC.00299-07. 17562800 PMC1932543

[40] Leavitt A, Chmelnitsky I, Ofek I, Carmeli Y, Navon-Venezia S 2010 Plasmid pKpQIL encoding KPC-3 and TEM-1 confers carbapenem resistance in an extremely drug-resistant epidemic *Klebsiella pneumoniae* strain. J Antimicrob Chemother 65:243–248. doi:10.1093/jac/dkp417. 19939824

[41] Getino M, de la Cruz F 2018 Natural and artificial strategies to control the. conjugative transmission of plasmids. Microbiol Spectr 6:MTBP-0015-2016. doi:10.1128/microbiolspec.MTBP-0015-2016. PMC11633558 29327679

[42] Buckner MMC, Ciusa ML, Piddock LV 2018 Strategies to combat antimicrobial resistance: anti-plasmid and plasmid curing. FEMS Microbiol Rev 42:781–804. doi:10.1093/femsre/fuy031. 30085063 PMC6199537

[43] Spengler G, Molnár A, Schelz Z, Amaral L, Sharples D, Molnár J 2006 The mechanism of plasmid curing in bacteria. Curr Drug Targets 7:823–841. doi:10.2174/138945006777709601. 16842214

[44] Dastidar S, Kristiansen J, Molnar J, Amaral L 2013 Role of phenothiazines and structurally similar compounds of plant origin in the fight against infections by drug resistant bacteria. Antibiotics (Basel) 2:58–72. doi:10.3390/antibiotics2010058. 27029292 PMC4790298

[45] Amábile Cuevas CF 1988 Loss of penicillinase plasmids of *Staphylococcus aureus* after treatment with L-ascorbic acid. Mutat Res 207:107–109. doi:10.1016/0165-7992(88)90072-3. 3258647

[46] Amábile-Cuevas CF, Piña-Zentella RM, Wah-Laborde ME 1991 Decreased resistance to antibiotics and plasmid loss in plasmid-carrying strains of *Staphylococcus aureus* treated with ascorbic acid. Mutat Res Lett 264:119–125. doi:10.1016/0165-7992(91)90128-Q. 1944394

[47] Ramesh A, Halami PM, Chandrashekar A 2000 Ascorbic acid-induced loss of a pediocin-encoding plasmid in *Pediococcus acidilactici* CFR K7. World J Microbiol Biotechnol 16:695–697. doi:10.1023/A:1008958517001.

[48] Fernandez-Lopez R, Machon C, Longshaw CM, Martin S, Molin S, Zechner EL, Espinosa M, Lanka E, de la Cruz F 2005 Unsaturated fatty acids are inhibitors of bacterial conjugation. Microbiology 151:3517–3526. doi:10.1099/mic.0.28216-0. 16272375

[49] Ripoll-Rozada J, García-Cazorla Y, Getino M, Machón C, Sanabria-Ríos D, de la Cruz F, Cabezón E, Arechaga I 2016 Type IV traffic ATPase TrwD as molecular target to inhibit bacterial conjugation. Mol Microbiol 100:912–921. doi:10.1111/mmi.13359. 26915347 PMC4908816

[50] Totsika M, Beatson SA, Sarkar S, Phan M-D, Petty NK, Bachmann N, Szubert M, Sidjabat HE, Paterson DL, Upton M, Schembri MA 2011 Insights into a multidrug resistant *Escherichia coli* pathogen of the globally disseminated ST131 lineage: genome analysis and virulence mechanisms. PLoS One 6:e26578. doi:10.1371/journal.pone.0026578. 22053197 PMC3203889

[51] Forde BM, Ben Zakour NL, Stanton-Cook M, Phan M-D, Totsika M, Peters KM, Chan KG, Schembri MA, Upton M, Beatson SA 2014 The complete genome sequence of *Escherichia coli* EC958: a high quality reference sequence for the globally disseminated multidrug resistant *E. coli* O25b:H4-ST131 clone. PLoS One 9:e104400. doi:10.1371/journal.pone.0104400. 25126841 PMC4134206

[52] McNally A, Oren Y, Kelly D, Pascoe B, Dunn S, Sreecharan T, Vehkala M, Välimäki N, Prentice MB, Ashour A, Avram O, Pupko T, Dobrindt U, Literak I, Guenther S, Schaufler K, Wieler LH, Zhiyong Z, Sheppard SK, McInerney JO, Corander J 2016 Combined analysis of variation in core, accessory and regulatory genome regions provides a super-resolution view into the evolution of bacterial populations. PLoS Genet 12:e1006280. doi:10.1371/journal.pgen.1006280. 27618184 PMC5019451

[53] Fookes M, Yu J, De Majumdar S, Thomson N, Schneiders T 2013 Genome sequence of Klebsiella pneumoniae Ecl8, a reference strain for targeted genetic manipulation. Genome Announc 1:e00027-12. doi:10.1128/genomeA.00027-12. PMC3569361 23405357

[54] De Majumdar S, Yu J, Fookes M, McAteer SP, Llobet E, Finn S, Spence S, Monahan A, Monaghan A, Kissenpfennig A, Ingram RJ, Bengoechea J, Gally DL, Fanning S, Elborn JS, Schneiders T 2015 Elucidation of the RamA regulon in *Klebsiella pneumoniae* reveals a role in LPS regulation. PLoS Pathog 11:e1004627. doi:10.1371/journal.ppat.1004627. 25633080 PMC4310594

[55] Phan MD, Forde BM, Peters KM, Sarkar S, Hancock S, Stanton-Cook M, Ben Zakour NL, Upton M, Beatson SA, Schembri MA 2015 Molecular characterization of a multidrug resistance IncF plasmid from the globally disseminated *Escherichia coli* ST131 clone. PLoS One 10:e0122369. doi:10.1371/journal.pone.0122369. 25875675 PMC4398462

[56] Hautefort I, Proenca MJ, Hinton J 2003 Single-copy green fluorescent protein gene fusions allow accurate measurement of Salmonella gene expression *in vitro* and during infection of mammalian cells. Appl Environ Microbiol 69:7480–7491. doi:10.1128/aem.69.12.7480-7491.2003. 14660401 PMC310007

[57] Arango Pinedo C, Smets BF 2005 Conjugal TOL transfer from *Pseudomonas putida* to *Pseudomonas aeruginosa*: effects of restriction proficiency, toxicant exposure, cell density ratios, and conjugation detection method on observed transfer efficiencies. Appl Environ Microbiol 71:51–57. doi:10.1128/AEM.71.1.51-57.2005. 15640169 PMC544212

[58] Sistrom WR 1977 Transfer of chromosomal genes mediated by plasmid r68.45 in *Rhodopseudomonas sphaeroides*. J Bacteriol 131:526–532. doi:10.1128/JB.131.2.526-532.1977. 407213 PMC235461

[59] Fernandez-Astorga A, Muela A, Cisterna R, Iriberri J, Barcina I 1992 Biotic and abiotic factors affecting plasmid transfer in *Escherichia coli* strains. Appl Environ Microbiol 58:392–398. doi:10.1128/AEM.58.1.392-398.1992. 1539984 PMC195220

[60] Madsen JS, Burmolle M, Hansen LH, Sorensen SJ 2012 The interconnection between biofilm formation and horizontal gene transfer. FEMS Immunol Med Microbiol 65:183–195. doi:10.1111/j.1574-695X.2012.00960.x. 22444301

[61] Krol JE, Nguyen HD, Rogers LM, Beyenal H, Krone SM, Top EM 2011 Increased transfer of a multidrug resistance plasmid in *Escherichia coli* biofilms at the air-liquid interface. Appl Environ Microbiol 77:5079–5088. doi:10.1128/AEM.00090-11. 21642400 PMC3147451

[62] Krol JE, Wojtowicz AJ, Rogers LM, Heuer H, Smalla K, Krone SM, Top EM 2013 Invasion of *E. coli* biofilms by antibiotic resistance plasmids. Plasmid 70:110–119. doi:10.1016/j.plasmid.2013.03.003. 23558148 PMC3687034

[63] Grimsey EM, Piddock LV 2019 Do phenothiazines possess antimicrobial and efflux inhibitory properties? FEMS Microbiol Rev 43:577–590. doi:10.1093/femsre/fuz017. 31216574

[64] Abdelmagid SA, Clarke SE, Nielsen DE, Badawi A, El-Sohemy A, Mutch DM, Ma D 2015 Comprehensive profiling of plasma fatty acid concentrations in young healthy Canadian adults. PLoS One 10:e0116195. doi:10.1371/journal.pone.0116195. 25675440 PMC4326172

[65] Sera RK, McBride JH, Higgins SA, Rodgerson DO 1994 Evaluation of reference ranges for fatty acids in serum. J Clin Lab Anal 8:81–85. doi:10.1002/jcla.1860080205. 8189326

[66] Padayatty SJ, Sun H, Wang Y, Riordan HD, Hewitt SM, Katz A, Wesley RA, Levine M 2004 Vitamin C pharmacokinetics: implications for oral and intravenous use. Ann Intern Med 140:533–537. doi:10.7326/0003-4819-140-7-200404060-00010. 15068981

[67] Elwell LP, Ferone R, Freeman GA, Fyfe JA, Hill JA, Ray PH, Richards CA, Singer SC, Knick VB, Rideout JL 1987 Antibacterial activity and mechanism of action of 3′-azido-3′-deoxythymidine (BW A509U). Antimicrob Agents Chemother 31:274–280. doi:10.1128/aac.31.2.274. 3551832 PMC174705

[68] Doleans-Jordheim A, Bergeron E, Bereyziat F, Ben-Larbi S, Dumitrescu O, Mazoyer M-A, Morfin F, Dumontet C, Freney J, Jordheim LP 2011 Zidovudine (AZT) has a bactericidal effect on enterobacteria and induces genetic modifications in resistant strains. Eur J Clin Microbiol Infect Dis 30:1249–1256. doi:10.1007/s10096-011-1220-3. 21494911

[69] Ng SMS, Sioson JSP, Yap JM, Ng FM, Ching HV, Teo JWP, Jureen R, Hill J, Chia C 2018 Repurposing zidovudine in combination with tigecycline for treating carbapenem-resistant *Enterobacteriaceae* infections. Eur J Clin Microbiol Infect Dis 37:141–148. doi:10.1007/s10096-017-3114-5. 29019016

[70] Peyclit L, Baron SA, Yousfi H, Rolain J-M 2018 Zidovudine: a salvage therapy for *mcr-1* plasmid-mediated colistin-resistant bacterial infections? Int J Antimicrob Agents 52:11–13. doi:10.1016/j.ijantimicag.2018.03.012. 29580929

[71] Lin Y-W, Abdul Rahim N, Zhao J, Han M-L, Yu HH, Wickremasinghe H, Chen K, Wang J, Paterson DL, Zhu Y, Rao GG, Zhou QT, Forrest A, Velkov T, Li J 2019 Novel polymyxin combination with the antiretroviral zidovudine exerts synergistic killing against NDM-producing multidrug-resistant *Klebsiella pneumoniae*. Antimicrob Agents Chemother 63:e02176-18. doi:10.1128/AAC.02176-18. 30670431 PMC6437486

[72] ViiV Healthcare. 2018 Retrovir—product monograph. ViiV Healthcare, Research Triangle, NC.

[73] Sandrini MPB, Clausen AR, On SLW, Aarestrup FM, Munch-Petersen B, Piskur J 2007 Nucleoside analogues are activated by bacterial deoxyribonucleoside kinases in a species-specific manner. J Antimicrob Chemother 60:510–520. doi:10.1093/jac/dkm240. 17615154

[74] Yssel AEJ, Vanderleyden J, Steenackers HP 2017 Repurposing of nucleoside- and nucleobase-derivative drugs as antibiotics and biofilm inhibitors. J Antimicrob Chemother 72:2156–2170. doi:10.1093/jac/dkx151. 28575223

[75] Lopatkin AJ, Huang S, Smith RP, Srimani JK, Sysoeva TA, Bewick S, Karig DK, You L 2016 Antibiotics as a selective driver for conjugation dynamics. Nat Microbiol 1:16044. doi:10.1038/nmicrobiol.2016.44. 27572835 PMC5010019

[76] Schuurmans JM, van Hijum S, Piet JR, Händel N, Smelt J, Brul S, ter Kuile BH 2014 Effect of growth rate and selection pressure on rates of transfer of an antibiotic resistance plasmid between *E. coli* strains. Plasmid 72:1–8. doi:10.1016/j.plasmid.2014.01.002. 24525238

[77] de Mol NJ, Busker RW 1984 Irreversible binding of the chlorpromazine radical cation and of photoactivated chlorpromazine to biological macromolecules. Chem Biol Interact 52:79–92. doi:10.1016/0009-2797(84)90084-x. 6209025

[78] Viola G, Latterini L, Vedaldi D, Aloisi GG, Dall’Acqua F, Gabellini N, Elisei F, Barbafina A 2003 Photosensitization of DNA strand breaks by three phenothiazine derivatives. Chem Res Toxicol 16:644–651. doi:10.1021/tx025680t. 12755594

[79] Plenge-Tellechea F, Domínguez-Solís CA, Díaz-Sánchez ÁG, Meléndez-Martínez D, Vargas-Medrano J, Sierra-Fonseca JA 2018 Chlorpromazine and dimethyl sulfoxide modulate the catalytic activity of the plasma membrane Ca(2+)-ATPase from human erythrocyte. J Bioenerg Biomembr 50:59–69. doi:10.1007/s10863-017-9741-9. 29313294

[80] Jiang Y-W, Gao G, Chen Z, Wu F-G 2017 Fluorescence studies on the interaction between chlorpromazine and model cell membranes. New J Chem 41:4048–4057. doi:10.1039/C7NJ00037E.

[81] Maruoka N, Murata T, Omata N, Takashima Y, Tanii H, Yonekura Y, Fujibayashi Y, Wada Y 2007 Effects of chlorpromazine on plasma membrane permeability and fluidity in the rat brain: a dynamic positron autoradiography and fluorescence polarization study. Prog Neuropsychopharmacol Biol Psychiatry 31:178–186. doi:10.1016/j.pnpbp.2006.08.019. 17023107

[82] Dabbeni-Sala F, Palatini P 1990 Mechanism of local anesthetic effect. Involvement of F0 in the inhibition of mitochondrial ATP synthase by phenothiazines. Biochim Biophys Acta 1015:248–252. doi:10.1016/0005-2728(90)90027-2. 2137014

[83] Bhattacharyya D, Sen PC 1999 The effect of binding of chlorpromazine and chloroquine to ion transporting ATPases. Mol Cell Biochem 198:179–185. doi:10.1023/a:1006902031255. 10497894

[84] Fronzes R, Christie PJ, Waksman G 2009 The structural biology of type IV secretion systems. Nat Rev Microbiol 7:703–714. doi:10.1038/nrmicro2218. 19756009 PMC3869563

[85] Christie PJ, Vogel JP 2000 Bacterial type IV secretion: conjugation systems adapted to deliver effector molecules to host cells. Trends Microbiol 8:354–360. doi:10.1016/s0966-842x(00)01792-3. 10920394 PMC4847720

[86] Daluge SM, Good SS, Faletto MB, Miller WH, St Clair MH, Boone LR, Tisdale M, Parry NR, Reardon JE, Dornsife RE, Averett DR, Krenitsky TA 1997 1592U89, a novel carbocyclic nucleoside analog with potent, selective anti-human immunodeficiency virus activity. Antimicrob Agents Chemother 41:1082–1093. doi:10.1128/AAC.41.5.1082. 9145874 PMC163855

[87] Faletto MB, Miller WH, Garvey EP, St Clair MH, Daluge SM, Good SS 1997 Unique intracellular activation of the potent anti-human immunodeficiency virus agent 1592U89. Antimicrob Agents Chemother 41:1099–1107. doi:10.1128/AAC.41.5.1099. 9145876 PMC163857

[88] Greig SL, Deeks ED 2015 Abacavir/dolutegravir/lamivudine single-tablet regimen: a review of its use in HIV-1 infection. Drugs 75:503–514. doi:10.1007/s40265-015-0361-6. 25698454

[89] Lewin CS, Allen RA, Amyes SG 1990 Mechanisms of zidovudine resistance in bacteria. J Med Microbiol 33:235–238. doi:10.1099/00222615-33-4-235. 2124270

[90] Salmon D, Detruchis P, Leport C, Bouvet E, Karam D, Meyohas MC, Coulaud JP, Vilde JL 1991 Efficacy of zidovudine in preventing relapses of Salmonella bacteremia in AIDS. J Infect Dis 163:415–416. doi:10.1093/infdis/163.2.415. 1988528

[91] Maier L, Pruteanu M, Kuhn M, Zeller G, Telzerow A, Anderson EE, Brochado AR, Fernandez KC, Dose H, Mori H, Patil KR, Bork P, Typas A 2018 Extensive impact of non-antibiotic drugs on human gut bacteria. Nature 555:623–628. doi:10.1038/nature25979. 29555994 PMC6108420

[92] Getino M, Fernández-López R, Palencia-Gándara C, Campos-Gómez J, Sánchez-López JM, Martínez M, Fernández A, de la Cruz F 2016 Tanzawaic acids, a chemically novel set of bacterial conjugation inhibitors. PLoS One 11:e0148098. doi:10.1371/journal.pone.0148098. 26812051 PMC4727781

[93] Getino M, Sanabria-Ríos DJ, Fernández-López R, Campos-Gómez J, Sánchez-López JM, Fernández A, Carballeira NM, de la Cruz F 2015 Synthetic fatty acids prevent plasmid-mediated horizontal gene transfer. mBio 6:e01032-15. doi:10.1128/mBio.01032-15. 26330514 PMC4556808

[94] Saw HTH, Webber MA, Mushtaq S, Woodford N, Piddock LV 2016 Inactivation or inhibition of AcrAB-TolC increases resistance of carbapenemase-producing *Enterobacteriaceae* to carbapenems. J Antimicrob Chemother 71:1510–1519. doi:10.1093/jac/dkw028. 26945714

[95] Cottell JL 2012 Investigation of factors influencing the successful persistence and dissemination of a globally distributed antibiotic resistance plasmid. University of Birmingham, Edgbaston, United Kingdom.

[96] Chan W, Costantino N, Li R, Lee SC, Su Q, Melvin D, Court DL, Liu P 2007 A recombineering based approach for high-throughput conditional knockout targeting vector construction. Nucleic Acids Res 35:e64. doi:10.1093/nar/gkm163. 17426124 PMC1885671

[97] Kim J, Webb AM, Kershner JP, Blaskowski S, Copley SD 2014 A versatile and highly efficient method for scarless genome editing in *Escherichia coli* and *Salmonella enterica*. BMC Biotechnol 14:84. doi:10.1186/1472-6750-14-84. 25255806 PMC4236582

[98] Sørensen SJ, Sørensen AH, Hansen LH, Oregaard G, Veal D 2003 Direct detection and quantification of horizontal gene transfer by using flow cytometry and gfp as a reporter gene. Curr Microbiol 47:129–133. doi:10.1007/s00284-002-3978-0. 14506860

[99] Musovic S, Oregaard G, Kroer N, Sørensen SJ 2006 Cultivation-independent examination of horizontal transfer and host range of an IncP-1 plasmid among gram-positive and gram-negative bacteria indigenous to the barley rhizosphere. Appl Environ Microbiol 72:6687–6692. doi:10.1128/AEM.00013-06. 17021220 PMC1610302

