# Supplementary material for: HIV Drugs Inhibit Transfer of Plasmids Carrying Extended-Spectrum β-Lactamase and Carbapenemase Genes
Source: mBio. 2020 Feb 25;11(1):e03355-19. doi: 10.1128/mBio.03355-19 (PMC7042701; doi:10.1128/mBio.03355-19)
Supplement: TEXT S1 [file mBio.03355-19-s0001.docx]

**Supplemental Text:**

**Results:**

*Confirmation of pEC958 curing from EC958*

Curing was confirmed by plasmid gel electrophoresis (Figure S1a). Minimum inhibitory concentrations (MICs) of kanamycin, ampicillin and cefotaxime, the resistance genes for which are encoded on pEC958, were lower for the cured strain (Table 1). PCR of regions of pEC958 and the curing plasmid, pCURE, confirmed loss of both plasmids (Table 1). Together this confirmed that the resulting strain, called ST131c, was free of both the curing plasmid pCURE and pEC958.

*Whole genome sequencing confirms strain identity*

Whole genome sequencing (WGS) confirmed that all *E. coli* strains belonged to MLST 131. ST131 EC958 contained IncFII and IncFIA replicons, consistent with pEC958.(1) ST131c and ST131c *mcherry* did not contain any plasmid replicons, and ST131c pCT*gfp* contained a replicon belonging to IncB/O/K/Z, which is consistent with the IncK replicon of pCT. Multiple drug-resistance genes were identified in ST131 EC958, including *bla*_CTX-M-14_, *bla*_TEM-1B_*, bla*_OXA-1_, *sul1,* and *tet(A).* These genes were all absent from the ST131c strain, consistent with plasmid curing. Both ST131 pCT*gfp* and *mcherry* contained the *aph* gene, which was used for cloning purposes. Comparison of the sequencing data for ST131 EC958 strain (which contains pEC958) with the published pEC958 sequence (HG941719) revealed three SNPs, one of which was located within the *tnpA1* gene (Table S4). Compared to the reference ST131 EC958 genome (NZ_HG941718.1), all of the ST131 EC958 strains (ST131 EC958, ST131c, ST131c pCT*gfp*, and ST131c *mcherry*) contained 33 SNPs (Table S5). In addition, there were SNPs unique to each strain (Table S5).

For the *E. coli* B104 strains, all three strains had the *ampC* β-lactamase gene and the PBP3 variant. B104/pCT*gfp* and B104 *mcherry* both contained the *aph(3’),* consistent with our cloning techniques. Sequencing confirmed the presence of the *gfp* gene in pCT in B104/pCT*gfp,* and the *mcherry* gene inserted into the *putPA* intergenic region in B104 *mcherry*.

The sequence data for pCT was compared with the published reference sequence (NC_014477.1) (2), which revealed nine SNPs (Table S6). Four SNPs in hypothetical proteins, four SNPs located within intergenic regions, and one SNP located in the *traH* conjugation machinery gene.

**Methods and Materials**

*Culture conditions, growth kinetics assays and MIC assays*

Bacteria were cultured in LB broth (Sigma-Aldrich, United Kingdom), and morpholinopropanesulfonic acid (MOPS) minimal medium supplemented with 0.2% glucose (Teknova). Antibiotics were added to media as needed at concentrations of 50 µg/mL kanamycin, 100 µg/mL rifampicin, 50 µg/mL tetracycline. API20E (BioMerieux), PCR, MacConkey’s selective agar, and Gram staining were routinely used to confirm strain identity. Bacterial strains and plasmids used in this study are listed in Table S5.

Bacterial growth during logarithmic phase was determined as described previously (3, 4). Briefly, an overnight culture was diluted to give a final inoculum of 0.1% (v/v) in 100 µl of fresh LB broth in a 96-well microtitre plate (Sterilin). Growth was monitored at OD_600_ at 10 min intervals per cycle for 100 cycles using the FLUOstar Optima plate reader (BMG Labtech). The bacterial generation times were calculated from the logarithmic phase and were determined using three technical replicates of three biological replicates, and were determined on three separate occasions. Comparisons between strains were performed using the Student’s *t*-test and was considered significantly different when p<0.05.

The MICs of antimicrobials, chlorpromazine (Sigma), linoleic acid (Sigma), ascorbic acid (Sigma) and abacavir (Sigma), and AZT (Sigma) were determined using the British Society of Antimicrobial Chemotherapy guidelines (5), and amended as necessary to be compliant with EUCAST recommended breakpoint concentrations (6). Consistent differences in the MIC of one doubling dilution lie within the error of the method and so were considered not significant.

*Curing of pEC958 from E. coli ST131 EC958*

*E. coli* ST131 EC958 was a gift from Dr. Peter A. Lund. We attempted to remove the resident large plasmid, pEC958, using a protocol outlined previously (7). However, despite numerous attempts we were unable to isolate successfully cured isolates using this protocol. Therefore, we used a plasmid developed by Dr. A. Lazdins and Prof. C.M. Thomas (Patent pending for plasmid), and successfully removed pEC958 from EC958. To ensure that the plasmid had been removed the MIC values of antimicrobials encoded on the plasmid (kanamycin, ampicillin, and cefotaxime) and chromosome (nalidixic acid) were determined for EC958 wildtype and EC958 cured (ST131c), as described above. Plasmid DNA extraction from EC958 wild type and ST131c was performed using QIAgen Plasmid Midi Kit (QIAGEN, cat. No. 12143) as per manufacturer’s protocol. Plasmid preparations (20 µL) were visualized using 0.7% agarose gel electrophoresis run at 50 volts at 4°C for 5 hours.

*Whole genome sequencing*

Strains ST131 EC958 (I1057), ST131c (I1067), ST131 pCT*gfp* (I1068), and ST131 *mcherry* (I1069) were whole genome sequenced by the Beijing Genomics Institute (BGI), using paired end sequencing carried out on the Illumina HiSeq 4000 platform. The Galaxy platform ([https://galaxyproject.org](https://galaxyproject.org/)) was used to perform FASTQ Grooming, QC, FASTQ Interlacing, and SPAdes in order to assemble genomes (8). Gene annotation was determined using PROKKA (<http://www.vicbioinformatics.com/software.prokka.shtml>) (9). The snippy program was used to search for SNPs (<https://github.com/tseemann/snippy>). The Center for Genomic Epidemiology platform (<http://www.genomicepidemiology.org/>) was used to determine the MLST (MLST-1.8) (10), plasmid incompatibility group (PlasmidFinder-1.3) (11), and to locate resistance genes (ResFinder-2.1) (12).

*Flow cytometry*

All flow cytometry was carried out using the Attune NxT Acoustic Focusing Flow Cytometer with Autosampler (Thermo Scientific), equipped with a blue/yellow (excitation laser – blue 488 nm, yellow 561 nm) laser configuration. Cells were illuminated with 488 nm (blue) and 561 nm (yellow) wavelength, data was collected using scatter, 530/30 filter (filter emission) and 620/15 filter (filter emission). For each sample, information on 10,000 bacterial cells was collected. Bacteria were gated based on GFP expression, mCherry expression, and the combined expression of both fluorescent proteins. Data was exported into Excel (Microsoft), and fluorescent populations were analysed using Student’s t-tests in GraphPad Prism.

*Determination of frequency of conjugation*

In order to perform classical conjugation assays, a rifampicin resistant variant of ST131c (ST131c^RifR^) was constructed by overnight growth on LB agar containing 100 µg/mL rifampicin. Candidate colonies were selected and growth kinetics were determined to ensure mutation did not impact growth rate. Sequencing of the *rpoB* gene was performed to identify the mutations resulting in rifampicin resistance. The frequency of mutation was calculated on three separate occasions and was 1.94x10^-10^ ± 7.86x10^-11^. For *K. pneumoniae* strain used is already resistant to rifampicin, therefore, a nalidixic acid resistant mutant was generated. This was done by selecting mutants of Ecl8 on LB agar plates containing 64 µg/mL nalidixic acid incubated at 37°C for 24 hours, then replica plating onto plates containing 128 µg/mL nalidixic acid at 37°C for 24 hours. Sequencing of PCR products revealed a S83F mutation in *gyrA,* which is previously associated with *K. pneumoniae* fluoroquinolone resistance (13). This Ecl8 Nal^R^ strain was used as the recipient for the classical conjugation assays.

Conjugation experiments were performed as described previously,(14) with the following modification: donors and recipients were co-incubated in LB broth in 96-well plates, to mimic the flow cytometry transmission assays. Bacteria were combined and incubated in 20 µg/mL chlorpromazine, 6 mM linoleic acid, 3.5 mg/mL ascorbic acid, 8 µg/mL abacavir, 0.008 μg/mL AZT as well as LB alone. After 24 hours incubation, samples were sub-cultured to agar plates containing kanamycin and rifampicin, or LB alone. After 24 hours incubation colonies were counted and velvet squares were used to replica plate from the LB plates onto either kanamycin or rifampicin plates. Conjugation frequencies were determined as previously (14).

**Supplemental Text References**

1. Phan MD, Forde BM, Peters KM, Sarkar S, Hancock S, Stanton-Cook M, Ben Zakour NL, Upton M, Beatson SA, Schembri MA. 2015. Molecular characterization of a multidrug resistance IncF plasmid from the globally disseminated *Escherichia coli* ST131 clone. PLoS One 10:e0122369.

2. Cottell JL, Webber MA, Coldham NG, Taylor DL, Cerdeño-Tárraga AM, Hauser H, Thomson NR, Woodward MJ, Piddock LJ V. 2011. Complete Sequence and Molecular Epidemiology of IncK Epidemic Plasmid Encoding *bla*(CTX-M-14). Emerg Infect Dis 17:645–652.

3. Webber MA, Randall LP, Cooles S, Woodward MJ, Piddock LJ V. 2008. Triclosan resistance in *Salmonella enterica* serovar Typhimurium. J Antimicrob Chemother 62:83–91.

4. Buckner MMC, Blair JMA, La Ragione RM, Newcombe J, Dwyer DJ, Ivens A, Piddock LJ V. 2016. Beyond Antimicrobial Resistance : Evidence for a Distinct Role of the AcrD Efflux Pump in *Salmonella* Biology. MBio 7:e01916-16.

5. Andrews JM. 2001. Determination of minimum inhibitory concentrations. J Antimicrob Chemother 48:5–16.

6. EUCAST. 2017. EUCAST Clinical Breakpoints. Eur Soc Clin Microbiol Infect Dis.

7. Schaufler K, Wieler LH, Semmler T, Ewers C, Guenther S. 2013. ESBL-plasmids carrying toxin-antitoxin systems can be “cured” of wild-type *Escherichia coli* using a heat technique. Gut Pathog 5:34.

8. Afgan E, Baker D, van den Beek M, Blankenberg D, Bouvier D, Čech M, Chilton J, Clements D, Coraor N, Eberhard C, Grüning B, Guerler A, Hillman-Jackson J, Von Kuster G, Rasche E, Soranzo N, Turaga N, Taylor J, Nekrutenko A, Goecks J. 2016. The Galaxy platform for accessible, reproducible and collaborative biomedical analyses: 2016 update. Nucleic Acids Res 44:W3–W10.

9. Seemann T. 2014. Prokka: rapid prokaryotic genome annotation. Bioinformatics 30:2068–2069.

10. Larsen M V, Cosentino S, Rasmussen S, Friis C, Hasman H, Marvig RL, Jelsbak L, Sicheritz-Ponten T, Ussery DW, Aarestrup FM, Lund O. 2012. Multilocus Sequence Typing of Total-Genome-Sequenced Bacteria. J Clin Microbiol 50:1355–1361.

11. Carattoli A, Zankari E, Garcia-Fernandez A, Voldby Larsen M, Lund O, Villa L, Moller Aarestrup F, Hasman H. 2014. In Silico Detection and Typing of Plasmids using PlasmidFinder and Plasmid Multilocus Sequence Typing. Antimicrob Agents Chemother 58:3895–3903.

12. Zankari E, Hasman H, Cosentino S, Vestergaard M, Rasmussen S, Lund O, Aarestrup FM, Larsen MV. 2012. Identification of acquired antimicrobial resistance genes. J Antimicrob Chemother 67:2640–2644.

13. Heiat M, Rezaeimehr MR, Moghaddam MM, Ranjbar R, Najafi A. 2014. Molecular genetic analysis of quinolone resistance-determining region of DNA Gyrase-A in fluoroquinolones resistant *Klebsiella pneumoniae* based on GenBank data and reported studies. Mol Genet Microbiol Virol 29:211–215.

14. Buckner MMC, Saw HTH, Osagie RN, McNally A, Ricci V, Wand ME, Woodford N, Ivens A, Webber MA, Piddock LJV. 2018. Clinically Relevant Plasmid-Host Interactions Indicate that Transcriptional and Not Genomic Modifications Ameliorate Fitness Costs of *Klebsiella pneumoniae* Carbapenemase-Carrying Plasmids. MBio 9:e02303-17.
