# Supplementary material for: HIV Drugs Inhibit Transfer of Plasmids Carrying Extended-Spectrum β-Lactamase and Carbapenemase Genes
Source: mBio. 2020 Feb 25;11(1):e03355-19. doi: 10.1128/mBio.03355-19 (PMC7042701; doi:10.1128/mBio.03355-19)
Supplement: FIG S1 [file mBio.03355-19-sf001.docx]

**
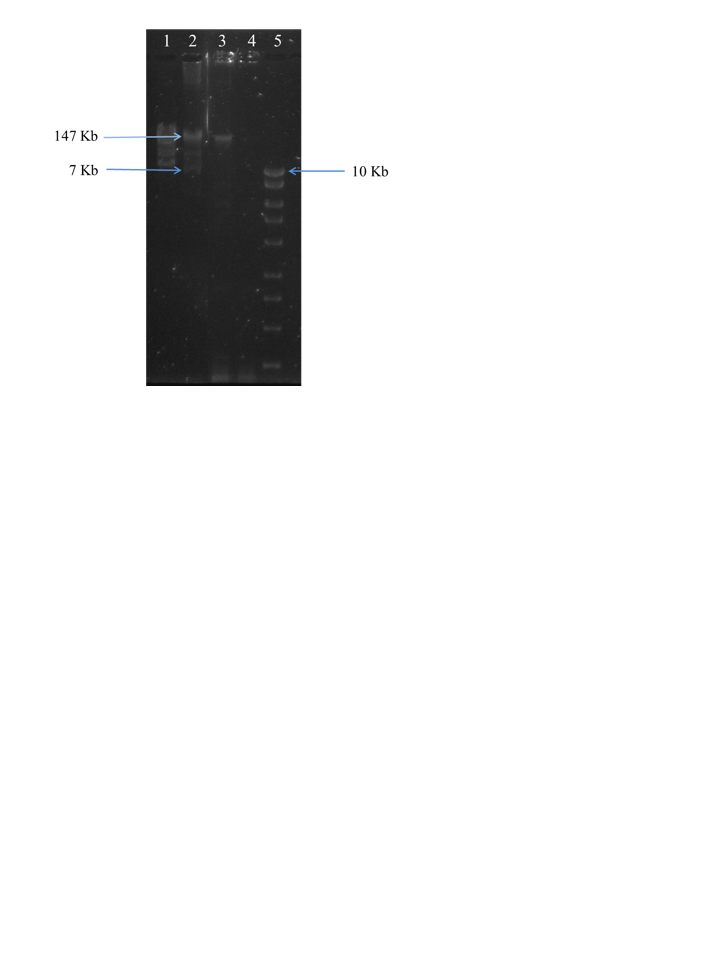
**

**(a)**

**(b)**

**Figure S1: (a)** Curing *E. coli* ST131 EC958 of plasmid pEC958. Lane 1: Hyperladder VI Bioline (band size from the top: 48.5, 38.42, 33.5, 29.95, 24.51, 23.99, 17.05, 15, 12.14, and 10.09 kb). Lane 2: *E. coli* reference strain NCTC 50192 containing 147, 63, 36 and 7 kb plasmids. Lane 3: *E. coli* ST131 EC958. Lane 4: *E. coli* ST131 EC958 cured of plasmid pEC958. Lane 5: Hyperladder 1kb Bioline (band size from the top: 10, 8, 6, 5, 4, 3, 2.5, 2 and 1.5 kb). **(b)** Quantification of cells co-localized, as percent of total cells in field of view, where co-localization is defined as pixels with both red and green signal, representing cells in very close contact, or where individual cell expresses both fluorescent proteins. Data from 3 independent experiments. Error bars represent standard deviation from the mean.
