# Supplementary material for: HIV Drugs Inhibit Transfer of Plasmids Carrying Extended-Spectrum β-Lactamase and Carbapenemase Genes
Source: mBio. 2020 Feb 25;11(1):e03355-19. doi: 10.1128/mBio.03355-19 (PMC7042701; doi:10.1128/mBio.03355-19)
Supplement: FIG S2 [file mBio.03355-19-sf002.docx]

**
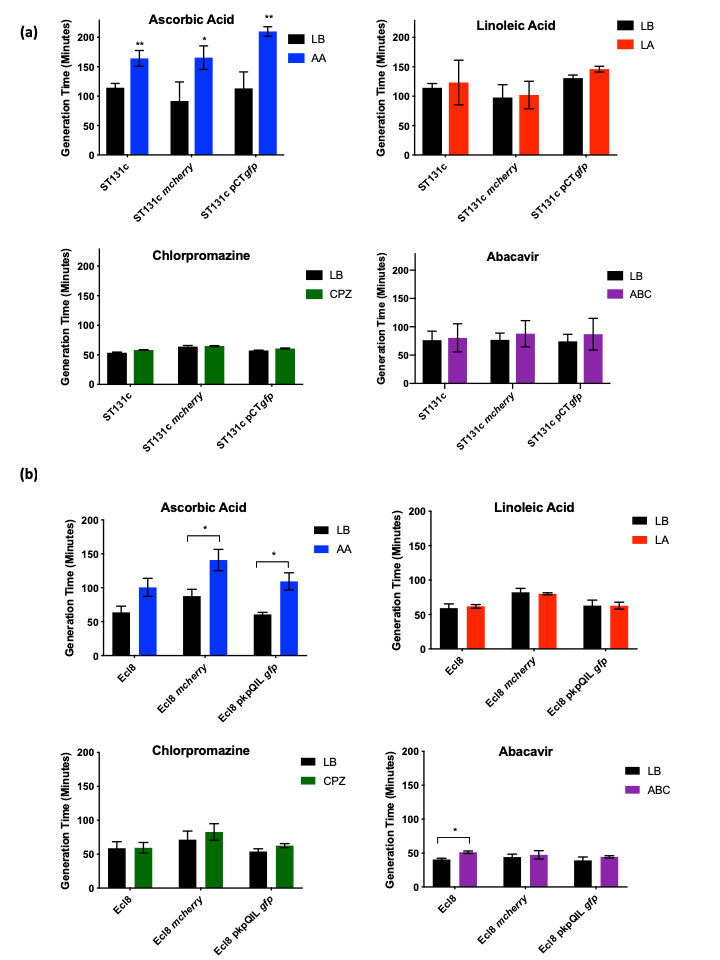
**

**
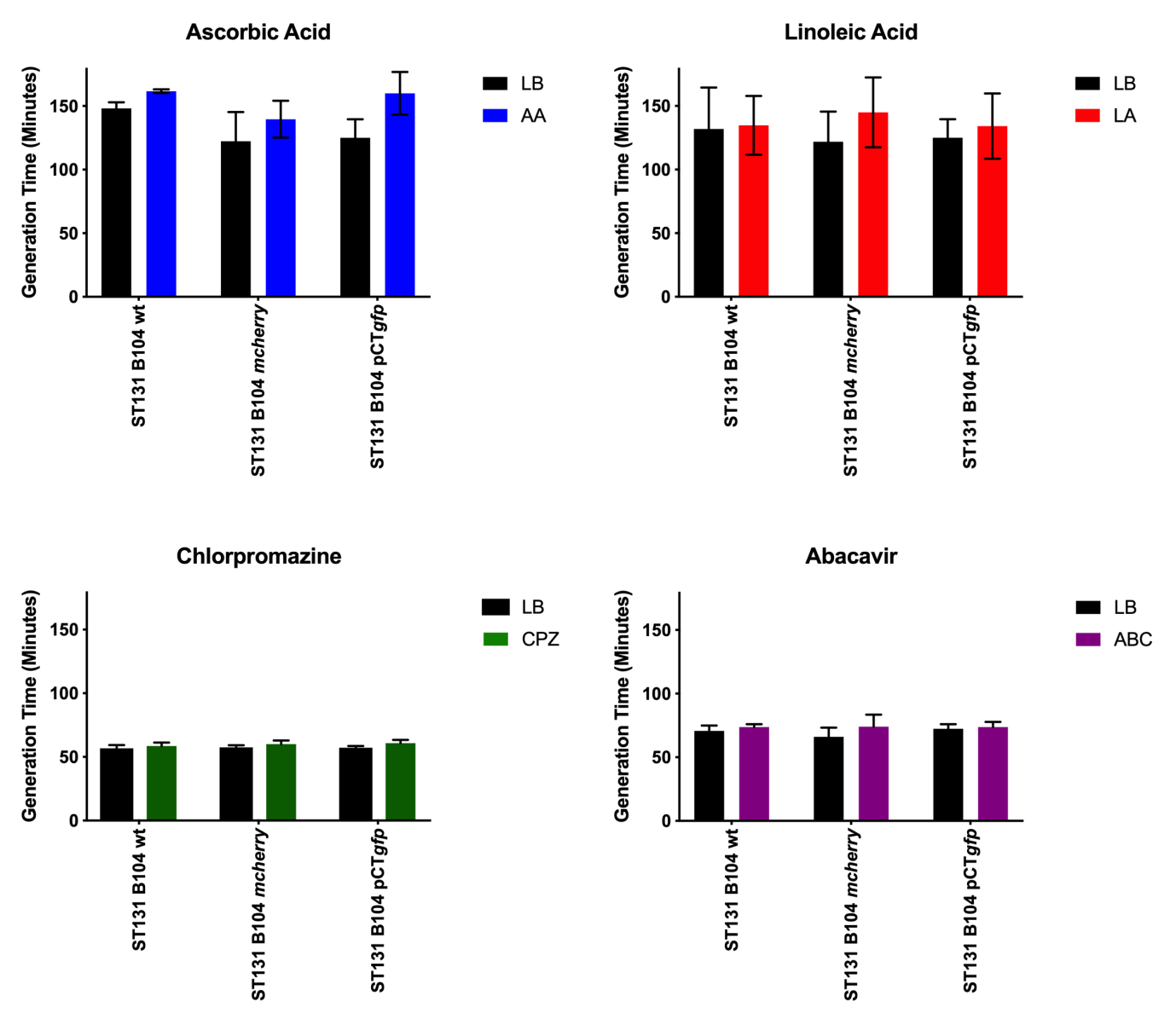
**

**(d)**

**(c)**

**Figure S2:** Generation time of bacteria in LB alone, or LB supplemented with either: 3.5 mg/mL ascorbic acid (blue), 6 mM linoleic acid (red), 20 µg/mL chlorpromazine (green), or 8 µg/mL abacavir (purple). **(a)** *E. coli* ST131 strains. **(b)** *K. pneumoniae* Ecl8 strains. **(c)** ST131 B104 strains. **(d)** Dose dependent effect of AZT on generation time of *E. coli* ST131c EC958 *mcherry* and pCT*gfp* strains and *K. pneumoniae mcherry* and pCT*gfp* strains. Data show the mean ± standard deviation of three independent experiments, comprised of at least three biological replicates. * denotes P<0.05, ** denotes P<0.001, n.s. denotes not significant.
