# Supplementary material for: HIV Drugs Inhibit Transfer of Plasmids Carrying Extended-Spectrum β-Lactamase and Carbapenemase Genes
Source: mBio. 2020 Feb 25;11(1):e03355-19. doi: 10.1128/mBio.03355-19 (PMC7042701; doi:10.1128/mBio.03355-19)
Supplement: FIG S3 [file mBio.03355-19-sf003.docx]

**
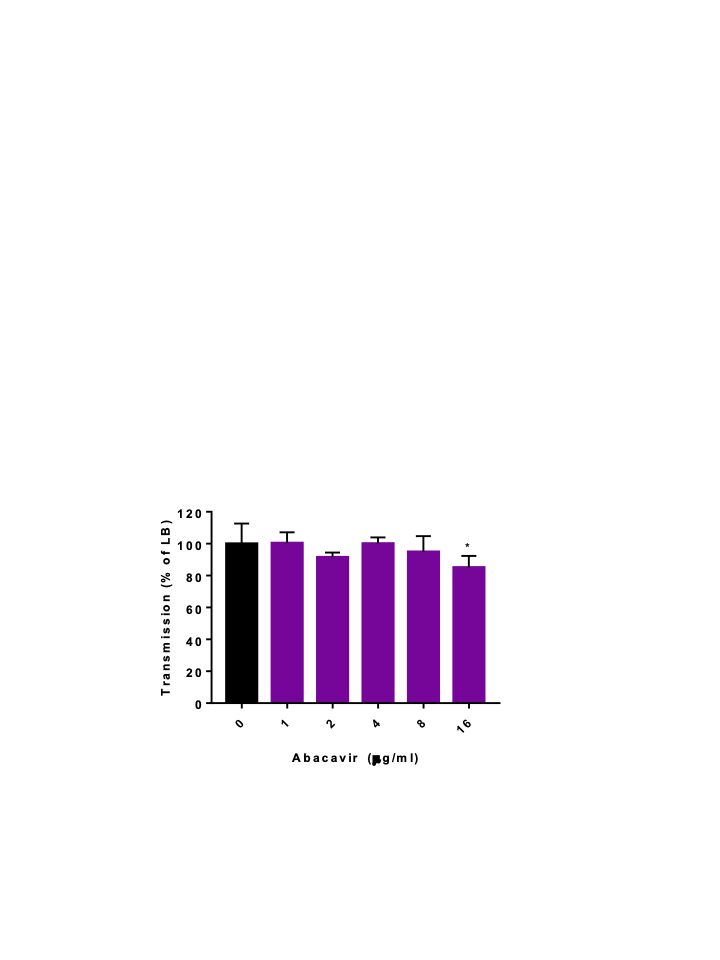
**

**Figure S3:** Impact of increasing concentrations of abacavir on pCT transmission in *E. coli* ST131 B104 strains. Data show mean ± standard deviation from three independent experiments, each with three biological replicates. * denotes P<0.05, ** denotes P<0.001.
