# Supplementary material for: HIV Drugs Inhibit Transfer of Plasmids Carrying Extended-Spectrum β-Lactamase and Carbapenemase Genes
Source: mBio. 2020 Feb 25;11(1):e03355-19. doi: 10.1128/mBio.03355-19 (PMC7042701; doi:10.1128/mBio.03355-19)
Supplement: TABLE S1 [file mBio.03355-19-st001.docx]

Table S1: Characteristics of abacavir and zidovudine (AZT).

| **ABACAVIR** | |
| --- | --- |
| **Category** | **Values and comments** |
| Drug-drug Interactions | Ethanol decreases elimination of abacavir (1).  Abacavir has no effect on the pharmokinetics of ethanol in males (1).  Abacavir increases oral methadone clearance by 22% (1). |
| Pharmacokinetics | **Absorption and Bioavailability in adults.** Administration of abacavir, in tablet form, leads to a geometric mean absolute bioavailability of 83% (1). An oral dose of 300 mg administered twice daily gives a steady-state peak serum abacavir concentration of 3.0±0.89 μg/ml (mean±SD) and AUC_(0-12 hr)_ of 6.02±1.73 μg*hr/mL (mean±SD) (1). A single 600 mg oral dose gives a steady-state peak serum concentrations of 4.26±1.19 μg/mL and AUC_∞_ of 11.95±2.51 μg*hr/mL (1). Values calculated from 20 subjects (1).  **Distribution.** The V_d_ of abacavir (150 mg IV administration) was 0.86±0.15 L/kg (proposing abacavir is distributed in extravascular space) (1). Abacavir bound to plasma proteins at ~50% and binding was not affected by concentration (1). CSF AUC_(0-6 hr)_ to plasma abacavir AUC _(0-6 hr)_ ratio was 27-33% in three subjects (1). Drug-related radioactivity concentration was identical for total blood and plasma (representative of abacavir distributing into erythrocytes) (1).  **Metabolism and Elimination.** The main route of abacavir elimination is through alcohol dehydrogenase and glucuronyl transferase. From a 600 mg dose of ^14^C-abacavir, 99% was recovered (assayed through radioactivity) (1). Abacavir was excreted in urine (1.2% of recovered), abacavir metabolites formed by alcohol dehydrogenase and glucuronyl transferase were also found in urine (66% of recovered), and the urine also contained unknown metabolites of abacavir (15% of total recovered) (1). 16% of the dose was eliminated through faeces (1). Observed elimination half-life (t_1/2_) was 1.54 ± 0.63 hours in single-dose trials (1). Total clearance was 0.80 ± 0.24 L/hr/kg (mean ± SD) after intravenous administration (1). |
| Toxicity | The oral LD_50_ of abacavir is >2000 mg/mL in rats (2)^.^  Rat studies demonstrate abacavir can be transported to the foetus via the placenta. At 500 mg/kg/day there is evidence for abacavir hemisulfate causing adverse maternal effects in rats (decreased length and foetal weight, increased observation of skeletal defects) (2). Although in rabbits there is no evidence of foetal malformation or teratogenicity in rabbits at 700 mg/kg/day (2).  Increased incidence of non- and malignant tumours when abacavir administered at 6 to 32 times the human dosage level in mice and rats (1).  Infants exposed to nucleoside reverse transcriptase inhibitors *in utero* have been reported to have mitochondrial dysfunction – main effects including haematological, metabolic and neurological disorders (3). |
| Contraindications | Previous hypersensitivity to abacavir (1).  Moderate or severe hepatic impairment (If given to those with mild impairment, patients should be monitored closely) (1, 3).  Avoid in end-stage renal disease (3). |
| Warnings/  precautions | Serious and occasionally fatal hypersensitivity associated with abacavir in ~8% of patients (patients with *HLA-B*5701* allele are at high risk) (1). Lactic acidosis and severe hepatomegaly with steatosis has been reported in patients undergoing alone or combinational antiretroviral treatment, including abacavir (sometimes fatally) (1). Immune reconstitution syndrome and redistribution/accumulation of body fat has been noted in patients receiving combination antiretroviral therapy, including abacavir (1). Higher risk of cardiovascular disease (myocardial infarctions) (3). Possible Stevens-Johnson syndrome (SJS) and toxic epidermal necrolysis (TEN) (1). |
| Adverse Reactions | Nausea, headache, malaise and fatigue, nausea and vomiting and dreams/sleep disorders (incidence ≥10%) (1). Fever and/or chills, nausea and vomiting, skin rashes, and ear/nose/throat infections (≥5%) (1).  **General side effects all nucleoside reverse transcriptase inhibitors:** “Abdominal pain, anaemia, anorexia, arthralgia, blood disorders, cough, diarrhoea, dizziness, dyspnoea, flatulence, gastro-intestinal disturbances, liver damage, metabolic effects, myalgia, neutropenia, osteonecrosis, pancreatitis, thrombocytopenia, and urticarial (Frequency of symptoms unknown)”(3). |
| Availability | The patent for the abacavir compound expired in 2010 (4). Patents on forms and formulations of abacavir however still exist, notably the hemisulfate salt (expires 2018) and the paediatric oral solution (expires 2019) (4). |
| Dose (1) | Tablets: 300 mg. Oral solution: 20 mg/ml  **Adults:** 300 mg, twice daily or 600 mg, once daily.  **Pediatric patients:** Aged 3 months and older (oral solution): 8mg/kg twice daily (up to 300 mg twice daily). 14 to 21 kg: 300 mg daily max. >21 to <30 kg: 450 mg daily max. ≥30 kg: 600 mg daily max  **Patients with Hepatic Impairment:** Mild hepatic impairment: 200 mg, twice daily. Moderate/severe hepatic impairment: contraindicated. |
| Cost (5) | Price for Ziagen (abacavir) on bnf for NHS as of (23/07/18)  **Oral solution:** 20 mg/ml, 240 ml for £55.72  **Tablets:** 300 mg, 60 tablets for £208.95 |
| Duration of dosing | Used to treat chronic HIV treatment. If dosing is stopped and then restarted, reintroduction of the drug is only recommended if medical care is available since in rare instances hypersensitivity is seen in patients with no prior hypersensitivity symptoms (1). |
| **ZIDOVUDINE (AZT)** | |
| Common names | Zidovudine, Azidothymidine, AZT, ZDV (6). |
| Molecular structure | C_10_H_13_N_5_O_4_  Molecular weight 267.245  IUPAC: 1-[(2R,4S,5S)-4-azido-5-(hydroxymethyl)oxolan-2-yl]-5-methyl-1,2,3,4-tetrahydropyrimidine-2,4-dione  Dideoxynucleoside compound with azido group substituted into sugar moiety (6). |
| Pharmacokinetics | **Absorption:** Rapid gastrointestinal absorption peaking at 0.5 – 1.5 hours, with bioavailability approximately 65% (7)  **Distribution:** Protein binding 30-38% with apparent volume of distribution = 1.6 +/- 0.6 L/kg. Peak serum concentrations range between 0.05-1.46 μg/mL (7).  **Metabolism:** Mean half-life from oral dose: 1 hour range (0.78-1.93). Inactivated by hepatic glucuronidating enzymes to 3'- azido-3'-deoxy-5'-O-β-D- glucopyranuronosylthymidine (GZDV), and 3’-amino-3’-deoxythymidine (AMT) by the cytochrome P450 system (6, 7).  **Excretion:** Renal (6). |
| Toxicity | Oral LD_50_ in male mice is 3084 mg/kg (6)  Adults ingesting large zidovudine quantities (10–22.5g) ‘experienced mild symptoms (drowsiness, lethargy, abdominal pain) and transient mild haematological abnormalities. … Drowsiness, convulsions and neutropenia have been reported after overdose*’* (8).  ^­^CHRONIC: *‘*Mitochondrial toxicity, sometimes fatal, has been associated with chronic use of NRTIs. This can manifest as peripheral neuropathy, myopathy, lipoatrophy, drug-induced pancreatitis and/or lactic acidosis with or without hepatic steatosis*’* (8). |
| Mechanism of action | A competitive nucleoside reverse transcriptase inhibitor causing suppression of HIV life-cycle. Specific mechanism inhibits conversion of ssRNA into dsDNA, a crucial preliminary step before integration into the host genome (6). |
| Clinical use | Suppression of viral load and disease progression in established HIV infection  Prevention of materno-foetal transmission of HIV (antenatal oral administration, intrapartum intravenous administration, oral solution administration to neonate) (7). |
| Dosing regimen | Typically 250-300 mg twice daily orally, in combination with other antiretroviral drugs  Can be administered intravenously (9). |
| Adverse Reactions | ‘anaemia, anxiety, chest pain, convulsions, depression, dizziness, drowsiness, gynaecomastia, influenza-like symptoms, lactic acidosis, lipodystrophy, loss of mental acuity, myopathy, neuropathy, paraesthesia, pigmentation of nails/skin/oral mucosa, pruritus, sweating, taste disturbance, urinary frequency’ (9).  General side effects of all nucleoside reverse transpritase inhibitors: ‘abdominal pain, anaemia, anorexia, arthralgia, blood disorders, cough, diarrhoea, dizziness, dyspnoea, fatigue, fever, flatulence, gastro-intestinal disturbances, headache, insomnia, liver damage, metabolic effects, myalgia, nausea, neutropenia, osteonecrosis, pancreatitis, rash, thrombocytopenia, urticaria, vomiting’ (9). |
| Contraindications | Abnormally low haemoglobin concentration, abnormally low neutrophil counts (9). |
| Cautions | The elderly, lactic acidosis, haematological toxicity (once stable requires 3 monthly monitoring of blood), Vitamin B12 deficiency (9). |
| Preparations | Available as: Intravenous solution, Oral solution, Oral capsules  Zidovudine 250 mg capsules (Aurobindo Pharma Ltd): £13.32 for 60 capsules (10). |

**Supplementary Table References:**

1. ViiVhealthcare. 2013. Full Perscribing Information- Ziagen.

2. GlaxoSmithKline. 2015. Safety data sheet- Ziagen Tablets, ViiVhealthcare.

3. BNF. British National Formulary - NICE, Abacavir. NICE.

4. UNITAID/World Health Organisation. 2014. 2014 Patents and licences on antiretrovirals : A snapshot.

5. BNF. British National Formulary - NICE, Abacavir- Medicinal Forms. NICE.

6. Drugbank.ca. 2018. Zidovudine, Drugbank. Drugbank.ca.

7. ViiVHealthcare. 2018. Retrovir - Product Monograph.

8. Toxbase.org. 2016. Toxbase, Zidovudine - UK National Poisons Informaion Service.

9. BNF. British National Formulary - NICE, Zidovudine.

10. BNF. British National Formulary - NICE, Zidovudine- Medicinal Forms.
