# Supplementary material for: HIV Drugs Inhibit Transfer of Plasmids Carrying Extended-Spectrum β-Lactamase and Carbapenemase Genes
Source: mBio. 2020 Feb 25;11(1):e03355-19. doi: 10.1128/mBio.03355-19 (PMC7042701; doi:10.1128/mBio.03355-19)
Supplement: TABLE S2 [file mBio.03355-19-st002.docx]

**Table S2** Mutation frequency of *E. coli* and *K. pneumoniae* strains to AZT.

| **Strain** | **Mutation Frequency** | **Standard Deviation** |
| --- | --- | --- |
| ST131c | 4.86x10^-7^ | 7.35x10^-8^ |
| ST131c pCT*gfp* | 5.52x10^-7^ | 2.69x10^-7^ |
| ST131c *mcherry* | 1.45x10^-6^ | 6.78x10^-7^ |
| *K. pneumoniae* Ecl8 | 3.97x10^-7^ | 2.18x10^-7^ |
| *K. pneumoniae* Ecl8 pKpQIL*gfp* | 8.65x10^-7^ | 6.77x10^-8^ |
| *K. pneumoniae* Ecl8 *mcherry* | 2.00x10^-7^ | 4.08x10^-8^ |

Data are averages of four biological replicates, with standard deviations from the means.
