# Supplementary material for: HIV Drugs Inhibit Transfer of Plasmids Carrying Extended-Spectrum β-Lactamase and Carbapenemase Genes
Source: mBio. 2020 Feb 25;11(1):e03355-19. doi: 10.1128/mBio.03355-19 (PMC7042701; doi:10.1128/mBio.03355-19)
Supplement: TABLE S3 [file mBio.03355-19-st003.docx]

**Table S3.** Bacterial strains, plasmids, and primers used in this study.

| **Strain Name** | | **Plasmid name and/or relevant genotype** | | **Description** | | **ARG code** | **Reference** | |  |
| --- | --- | --- | --- | --- | --- | --- | --- | --- | --- |
| *E.coli* BW25141 | | pKD4 | | *aph* gene | | I633 | (1) | |  |
| *S. enterica* SL1344 | | pUA66*pacpP* | | pUA66 carrying *gfp-mut2-aph* under *acpP* promoter | | L1019 | From P. Lund | |  |
| *E. coli* DH5α | | pCT_CTX-M-14_ | | E. coli containing pCT*bla*_CTX-M-14_ | | I755 | (2) | |  |
| *E.coli* DH5α | | pCT*gfp* | | pCT with *gfp-aph* inserted into *bla*_CTX-M-14_ | | I1003 | (3) | |  |
| *E. coli* DH5α | | pET17b p*acpP* *mcherry aph* | | *acpP* promoter inserted upstream of *mcherry*, with *aph* immediately downstream of *mcherry* | | I1055 | This work | |  |
| *E. coli* DH5α | | curing plasmid | | plasmid curing system | | I1056 | From C. Thomas | |  |
| *E. coli* ST131 EC958 | | pEC958 | | Wildtype EC958 | | I1057 | From P. Lund | |  |
| *E. coli* ST131c | | - | | ST131 EC958 cured of pEC958 | | I1067 | This work | |  |
| *E. coli* ST131c pCT*gfp* | | pCT*gfp* | | I1067 carrying pCT with *gfp aph* inserted into *bla*_CTX-M-14_ | | I1068 | This work | |  |
| *E. coli* ST131c pSIM18 | | pSIM18 | | I1067 carrying pSIM18 for recombineering | | I1073 | pSIM18 from (4) | |  |
| *E. coli* ST131c *mcherry* | | *mcherry* | | I1067 with *mcherry aph* inserted into chromosomal *putPA* intergenic region | | I1069 | This work | |  |
| *E. coli* ST131c Rif Resistant | | *rpoB* H537Y | | Rifampicin resistant strain | | I1113 | This work | |  |
| *E. coli* ST131 B104 | | - | | Naturally plasmid-free clinical isolate of ST131 clonal group | | I1109 | (5) | |  |
| *E. coli* ST131 B104 Rif resistant | | *rpoB* | | I1109 with mutation resulting in increased resistance to rifampicin | | I1125 | This work | |  |
| *E. coli* ST131 B104 pSLTS | | pSLTS | | I1109 carrying pSLTS for recombineering | | I1128 | pSLTS from (6) | |  |
| *E. coli* ST131 B104 *mcherry* | | *mcherry* | | I1125 with *mcherry aph* inserted into chromosomal *putPA* intergenic region | | I1138 | This work | |  |
| *E. coli* ST131 B104 *pCTgfp* | | *pCTgfp* | | I1125 carrying pCT with *gfp aph* inserted into *bla*_CTX-M-14_ | | I1126 | This work | |  |
| *K. pneumoniae* Ecl8 | | *rpoB* | | His537Leu substitution in *rpoB* resulting in resistance to rifampicin. | | H222 | (7) | |  |
| *E. coli* SW105 | | pKpQIL | | SW105 containing chromosomal recombinase, and pKpQIL | | I940 | This work | |  |
| *E. coli* DH10B | | - | | *E. coli* for cloning purposes. | | I910 | Invitrogen | |  |
| *E. coli* DH10B pKpQIL*gfp* | | pKpQIL*gfp* | | Constructed plasmid pKpQIL containing *gfp* insertion into KPC gene. | | I1019 | This work | |  |
| *K. pneumoniae* Ecl8 pKpQIL*gfp* | | pKpQIL*gfp* | | Ecl8 carrying pKpQIL*gfp* inserted into *bla*_KPC_ | | H235 | This work | |  |
| *K. pneumoniae* Ecl8 *mcherry* | | *mcherry* | | Ecl8 with *mcherry* inserted into chromosome at *putPA* intergenic region | | H234 | This work | |  |
| *K. pneumoniae* Ecl8 Nal^R^ | | *gyrA* | | Ser83Phe substitution in *gyrA* resulting in fluoroquinolone resistance. | | H237 | This work | |  |
| **Primer** | **Target** | | **Sequence (5’-3’)** | | **Description** | | | **Referen-ce** | |
| **819** | *bla*_CTX-M-14_ in pCT | | ATGGTGACAAAGAGAGTGCAA | | amplifies *bla*_CTX-M-14_ in pCT (and amplifies *gfp aph* in pCT*gfp*) | | | (8) | |
| **820** |  |  | TTACAGCCCTTCGGCGATG | |  |  |  |  |  |
| **2100** | *aph* gene on pKD4 | | agctagCTCGAGactggtgtaggctggagctgcttc | | amplifies *aph* with XhoI sites on either side from pKD4 | | | This work | |
| **2101** |  |  | gacttcCTCGAGagctGGGAATTAGCCATGGTCCATatga | |  |  |  |  |  |
| **2102** | *aph* gene on pET17b | | CTCATCCTGTCTCTTGATCAGATC | | check direction of *aph* after XhoI insertion (to be used with 2100 as F) | | | This work | |
| **2223** | Inserts *mcherry* into EC958 | | AATGTAAATGGTGTGTTAAATCGATTGTGAATAACCAGCGCTTCCTGGCGCAGGTTCCTGCGG | | amplifies the *pacpP mcherry aph* fragment from pET17b p*acpP-mcherry-aph* for integration into ST131 EC958 chromosome at *putPA* intergenic region | | | This work | |
| **2224** |  |  | CGAAACTTGCCGTTATATCTGCCACCGGAACGGGGTAACAGAGTTGCTGGGAATTAGCCATGGTCCATATG | |  |  |  |  |  |
| **2215** | *pacpP*-*mcherry-aph* in EC958 | | CGGCAGGTCTTACCACGATT | | Check for the insertion of *mcherry* in EC958 chromosome | | | This work | |
| **2225** |  |  | TCTCTGCGGCAGTTAACATT | |  |  |  |  |  |
| **2098** | *pacpP* for pET17b *mCherry* | | taattcAAGCTTatTGGCGCAGGTTCCTGCGGGTCGCCT | | amplifies *acp* promoter from pUA66*pacpP* with HinDIII and BamHI sites for ligation into pET17*mCherry* | | | This work | |
| **2099** |  |  | cacgtaGGATCCgtATCCGCGCCCAGGTCTTCAACGAAA | |  |  |  |  |  |
| **2301** | Inserts *mcherry* into ST131 B104 | | AGGGCGACCGTATCCTGCCGGAAGCGCTGGTTATTCACAATCGATTTAACACACCTGGCGCAGGTTCCTGCGG | | amplifies the *pacpP mcherry aph* fragment from pET17b p*acpP-mcherry-aph* for integration into ST131 B104 chromosome at *putPA* intergenic region | | | This work | |
| **2302** |  |  | TGCAACTTAACGTTATCGTGAAATATCCATGATGTTGCAACTTTGTGCAACCATGGCTGGGAATTAGCCATGGTCCATATG | |  |  |  | This work | |
| **2303** | *pacpP*-*mcherry-aph* in B104 | | gtcaccaacatcggtgtgcta | | Check for the insertion of *mcherry* in EC958 chromosome. Used with 2215 | | | This work | |
| **FIA Fw** | IncFIA | | ccatgctggttctagagaaggtg | | check for presence of IncFIA group plasmid | | | (9) | |
| **FIA Rev** |  |  | gtatatccttactggcttccgcag | |  |  |  |  |  |
| **AntiF Fw** | curing plasmid | | gtcgacgtcccctgttatccctacccgg | | check for presence of curing plasmid | | | A. Lazdins and C.M. Thomas | |
| **AntiF Rev** |  |  | gcgagatctagggtaatcccggatcttcg | |  |  |  |  |  |
| **2554** | Thymidine Kinase | | ATGATAAACTCCAGCCAACTTTATTTC | | Sequencing thymidine kinase | | | This work | |
| **2555** |  | | GTGTATAGCCCGGAAGAAGTG | |  | | |  | |
| **1551** | *aph-gfpmut2* | | CAACCTCGTCGCGGAACCATTCGCTAAACTCGAACAGGACTTTG(CAGGAGTCCAAGCGAGCTCT) | | Flanking primers to amplify the *aph-gfpmut2* cassette from pUA66. | | | This work | |
| **1552** |  | | GCCAGTGCAGAGCCCAGTGTCAGTTTTTGTAAGCTTTCCG(AGGAGAGCGTTCACCGACAA) | | Flanking primers to amplify the *aph-gfpmut2* cassette from pUA66. | | | This work | |
| **1501** | *bla*_KPC_ | | ATGTCACTGTATCGCCGTCT | | Primers to check *bla*_KPC_ gene | | | (7) | |
| **1502** |  | | TAGACGGCCAACACAATAGG | |  | | |  | |
| **2212** | *pacpP-mCherry-aph* | | tcggatcagggctggcattacaggatgaggagctatcaggaacgtTGGCGCAGGTTCCTGCGG | | Amplifies *pacpP-mCherry-aph* from pET17 to insert into Ecl-8 at 142582 | | | This work | |
| **2213** |  | | gtcctctacggcttttcttagcgcactgacactcctgaactccctgctGGGAATTAGCCATGGTCCATATG | |  | | |  | |
| **2214** | *mcherry* check | | Ccgaagctcagaccgggaga | | Amplifies 5' overlap after *mcherry* insertion into Ecl8 | | | This work | |
| **2215** |  | | CGGCAGGTCTTACCACGATT | |  | | |  | |
| **2012** | *gyrA* | | ATACAGTAGAGGGATAGCGG | | Amplifies first segment of Ecl8 *gyrA* | | | (10) | |
| **2377** |  | | GCGTCCACTTCCACTTCC | |  | | | This work | |
| **2378** | *gyrA* | | CCATTATCAATGGCCGTCGC | | Amplifies second segment of Ecl8 *gyrA* | | | This work | |
| **2017** |  | | TCAGTTCGATAATCGGGTCG | |  | | | (10) | |
| **2379** | *gyrA* | | CGTTGCGCTGGCCAACAT | | Amplifies third segment of Ecl8 *gyrA* | | | This work | |
| **2380** |  | | GACGGCTGAACTCGGTCA | |  | | | This work | |
| **2381** | *gyrA* | | AAGAGGGCGTCAACGTCTT | | Amplifies fourth segment of Ecl8 *gyrA* | | | This work | |
| **2382** |  | | CCTCCCTTCTGTGCGATAT | |  | | | This work | |

**Table S3 References:**

1. Datsenko KA, Wanner BL. 2000. One-step inactivation of chromosomal genes in *Escherichia coli* K-12 using PCR products. Proceedings of the National Academy of Sciences of the United States of America 97:6640–6645.

2. Cottell JL, Webber MA, Piddock LJ V. 2012. Persistence of Transferable Extended-Spectrum-β-Lactamase Resistance in the Absence of Antibiotic Pressure. Antimicrobial Agents and Chemotherapy 56:4703–4706.

3. Cottell JL. 2012. Investigation of factors influencing the successful persistence and dissemination of a globally distributed antibiotic resistance plasmid. University of Birmingham.

4. Chan W, Costantino N, Li R, Lee SC, Su Q, Melvin D, Court DL, Liu P. 2007. A recombineering based approach for high-throughput conditional knockout targeting vector construction. Nucleic Acids Research 35:e64–e64.

5. McNally A, Oren Y, Kelly D, Pascoe B, Dunn S, Sreecharan T, Vehkala M, Välimäki N, Prentice MB, Ashour A, Avram O, Pupko T, Dobrindt U, Literak I, Guenther S, Schaufler K, Wieler LH, Zhiyong Z, Sheppard SK, McInerney JO, Corander J. 2016. Combined Analysis of Variation in Core, Accessory and Regulatory Genome Regions Provides a Super-Resolution View into the Evolution of Bacterial Populations. PLOS Genetics 12:e1006280.

6. Kim J, Webb AM, Kershner JP, Blaskowski S, Copley SD. 2014. A versatile and highly efficient method for scarless genome editing in *Escherichia coli* and *Salmonella enterica*. BMC Biotechnology 14:84.

7. Saw HTH, Webber MA, Mushtaq S, Woodford N, Piddock LJ V. 2016. Inactivation or inhibition of AcrAB-TolC increases resistance of carbapenemase-producing *Enterobacteriaceae* to carbapenems. Journal of Antimicrobial Chemotherapy 71:1510–1519.

8. Bradley DE. 1984. Characteristics and function of thick and thin conjugative pili determined by transfer-derepressed plasmids of incompatibility groups I1, I2, I5, B, K and Z. Journal of general microbiology 130:1489–1502.

9. Carattoli A, Bertini A, Villa L, Falbo V, Hopkins KL, Threlfall EJ. 2005. Identification of plasmids by PCR-based replicon typing. Journal of microbiological methods 63:219–228.

10. Webber MA, Ricci V, Whitehead R, Patel M, Fookes M, Ivens A, Piddock LJ V. 2013. Clinically Relevant Mutant DNA Gyrase Alters Supercoiling, Changes the Transcriptome, and Confers Multidrug Resistance. mBio 4:e00273-13.
